# Toxicity Assessment of Biogenic Gold Nanoparticles on Crop Seeds and Zebrafish Embryos: Implications for Agricultural and Aquatic Ecosystems

**DOI:** 10.1021/acsomega.4c08287

**Published:** 2025-01-03

**Authors:** Caroline E. A. Botteon, Anderson do E. S. Pereira, Larissa P. de Castro, Isabela A. Justino, Leonardo F. Fraceto, Jairo K. Bastos, Priscyla D. Marcato

**Affiliations:** † School of Pharmaceutical Sciences of Ribeirão Preto, University of São Paulo, Ribeirão Preto 14440-903, Brazil; ‡ Institute of Science and Technology, São Paulo State University, Sorocaba 18087-180, Brazil

## Abstract

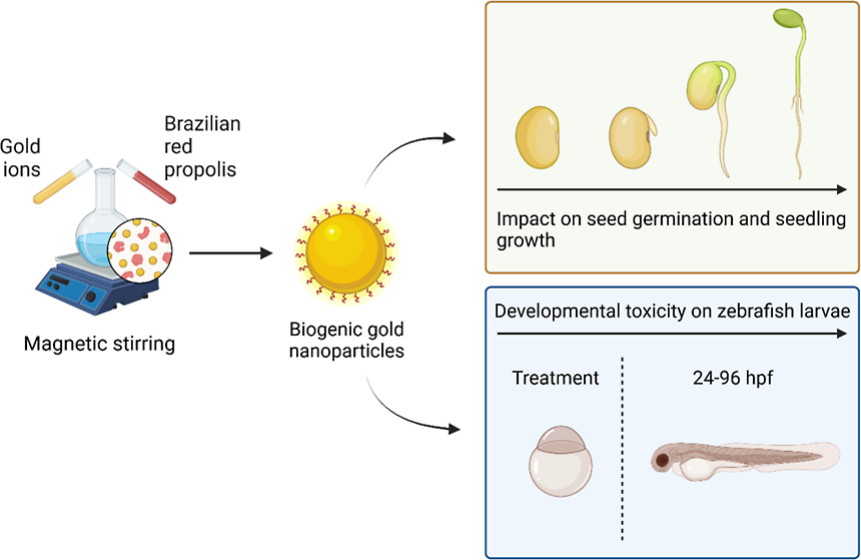

The demand for food production has been growing exponentially due to the increase in the global population. Innovative approaches to enhance agricultural productivity have been explored, including the new applications of nanoparticles in agriculture. The nanoparticle application in agriculture can generate environmental and human health risks since nanoparticles can contaminate the soil and inevitably reach groundwater, potentially causing toxicity in aquatic organisms. In this study, we evaluated the benefits and toxicity of gold nanoparticles (GNPs), synthesized via green chemistry, on the growth of cultivated plants and in the zebrafish embryo model. GNPs were synthesized through an economical and environmentally friendly method using Brazilian red propolis (BRP) extract (BRP-GNPs). BRP-GNPs exhibited negative and positive effects on plant germination, depending on the concentration tested and the plant species involved. Moreover, BRP-GNPs induced developmental toxicity in fish embryos in a dose-dependent manner. Our results provide valuable insights for assessing the environmental risks of biogenic GNPs.

## 1 Introduction

As the global population continues to rise, the demand for food production is increasing exponentially. Thus, ongoing research and innovation to improve crop production are necessary to overcome this challenge, including the relatively new application of nanoparticles in agriculture. Researchers have reported the capacity of several nanomaterials to improve the yields of different crops.^1^ Seeds are treated with nutrients, hormones, and pesticides in nanoscale to protect and enhance their useful properties under stressful environmental conditions.^2^ Prefarming seed treatments, including nanopriming and nanocoating, are considered efficient and cheap.^3^ Seed nanopriming is a method of soaking seeds in a dispersion containing nanoparticles and some nutrient or other additives for a period of time, after which the seeds are dried before being sown. In the nanocoating technique, a dispersion or nanoformulation is sprayed onto the seeds forming a homogeneous layer.^4^ Nutrient absorption efficacy, photosynthesis, nutrient translocation, and pathogen resistance are reported to be significantly improved with the use of this nanoagrochemical.^5^

Gold nanoparticles (GNPs) have been intensively used for several applications, such as catalysis, sensors, drug delivery, antitumor agents, antimicrobials, antioxidants, larvicides, and agriculture.^6,7^ Green-synthesized nanoparticles have been investigated in plant biological sciences.^8^ Chemically synthesized metallic nanoparticles are easily prepared and characterized; however, they generally demonstrate toxicity due to the chemical compounds used in the reaction.^9^ It is widely known that the surface coatings of nanoparticles play a crucial role in determining their activity and toxicity. As chemically synthesized nanoparticles usually use chemically substantial reducing and stabilizer agents, their toxicity will be partly determined by the interaction of these molecules with cells and other living organisms. Consequently, replacing these ligands with biocompatible molecules is a required step in systems intended for biological application.

On the other hand, nanoparticles synthesized by green methods (environmentally friendly methods) using plants, fungi, bacteria, and algae, among others, display the advantage of having molecules derived from their precursors as surface ligands and stabilizers, rendering them more biocompatible and less toxic.^10^ In addition, metallic nanoparticle production by green methods most often involves the use of nontoxic universal solvents, such as water, thereby minimizing waste generation. Thus, incorporating biogenic nanoparticles into agricultural operations, such as nanofertilizers, can improve the efficiency and stimulate sustainability in agricultural procedures. Parveen et al. (2016)^11^ reported that GNPs biosynthesized with *Cassia auriculata* leaf extract at room temperature positively affected the percentage of seed germination and growth of seedlings of *Pennisetum glaucum*.

Despite the known beneficial effects of nanoparticles in improving seed metabolism and stimulating plant growth, some nanomaterials can cause adverse effects on plants, such as inhibition of germination and seedling toxicity.^12^ After plant tissue exposure, nanoparticles migrate to the grown plantlets, accumulating in several tissues, including newly formed seeds. This seed germination generates the emergence of second-generation plantlets where nanoparticles are detected in the leaves.^13^ The use of materials in nanoscale in agricultural production nowadays is unprecedent; therefore, contamination of the environment is a possible risk that must be considered.^14^ Thereby, the emergence of new nanoparticle applications will increase the amount of nanosized materials released in the aquatic environment due to wastewater runoff from domestic and industrial sources or agricultural applications.^15,16^ In addition, there have been increasing remediation techniques for water and soil employing nanomaterials, expanding their use in the environmental area.^17^

Nanoagrochemical residues can remain in the soil or leach to groundwater, reaching aquifers.^18^ Once in aquifers, nanomaterials can access river systems through surface runoff and soil and aquifer infiltration.^18,19^ Nanoparticles can be absorbed by filter feeders and animals that live in sediments, leading to possible biomagnification in the food chain.^15^ Recent research indicates that smaller GNPs tend to be more ingested and bioaccumulated by organisms.^16^ Furthermore, Unrine et al. (2012)^20^ have investigated GNP trophic transfer, showing that GNPs were transferred from soil to invertebrates and then to secondary consumers, thus enhancing the extent of bioaccumulation and the effects of these nanoparticles when applied or released into the environment. Therefore, scientists are making a great effort to understand the toxicity of GNPs with different shapes, sizes, and attachments in aquatic organisms.^21^ The physicochemical properties of GNPs can influence the adsorption of molecules on their surface and the aggregation or sedimentation of nanoparticles. These processes may contribute to the uptake and toxic effects of GNPs in aquatic organisms.^16^

Despite the presence of a natural capping on the green synthesized nanoparticles, it does not guarantee any harmlessness to nontarget organisms.^22^ Previous studies have reported that different biosynthesized nanoparticles may cause toxicity by oxidative stress, through the generation of reactive oxygen species (ROS) (oxidative stress), cell membrane disruption, inhibition of the electron transport chain, inflammatory processes, and genotoxicity, which leads to DNA damage, chromosomal aberration, and mutations.^23,24^ The exact mechanism by which biogenic nanoparticles can cause toxic effects has not been established yet as there are limited available data about their metabolism, biological behavior, distribution, and bioaccumulation.^23,25^ Depending on the raw material used in the synthesis of GNPs, the resultant characteristics of the nanomaterials, as well as their toxicity, may be higher, lower, or nonexistent.^22^

The toxicologic effects of exposure to nanoparticles are a growing concern.^26,27^ Although there is wide evidence about the benefits of the use of nanoparticles in medicine and agriculture, the current understanding of the impact of the frequent exposure to nanoparticles on human health and the environment is still limited.^28,29^ Thus, investigating the toxicity of GNPs is crucial to evaluating potential risks and their impact on the ecosystem. In this context, this research aimed to investigate the pretreatment of seeds with biogenic GNPs synthesized from Brazilian red propolis (BRP), focusing on their potential positive and negative effects on the germination and growth of important crops. Additionally, the study sought to examine the toxic effects of GNPs on zebrafish embryos, contributing to the understanding of the impact that biogenic GNPs may have on agricultural practices and their subsequent effects on aquatic systems.

## 2 Results and Discussion

### 2.1 Synthesis and Characterization of BRP-GNPs

**Figure 1:**
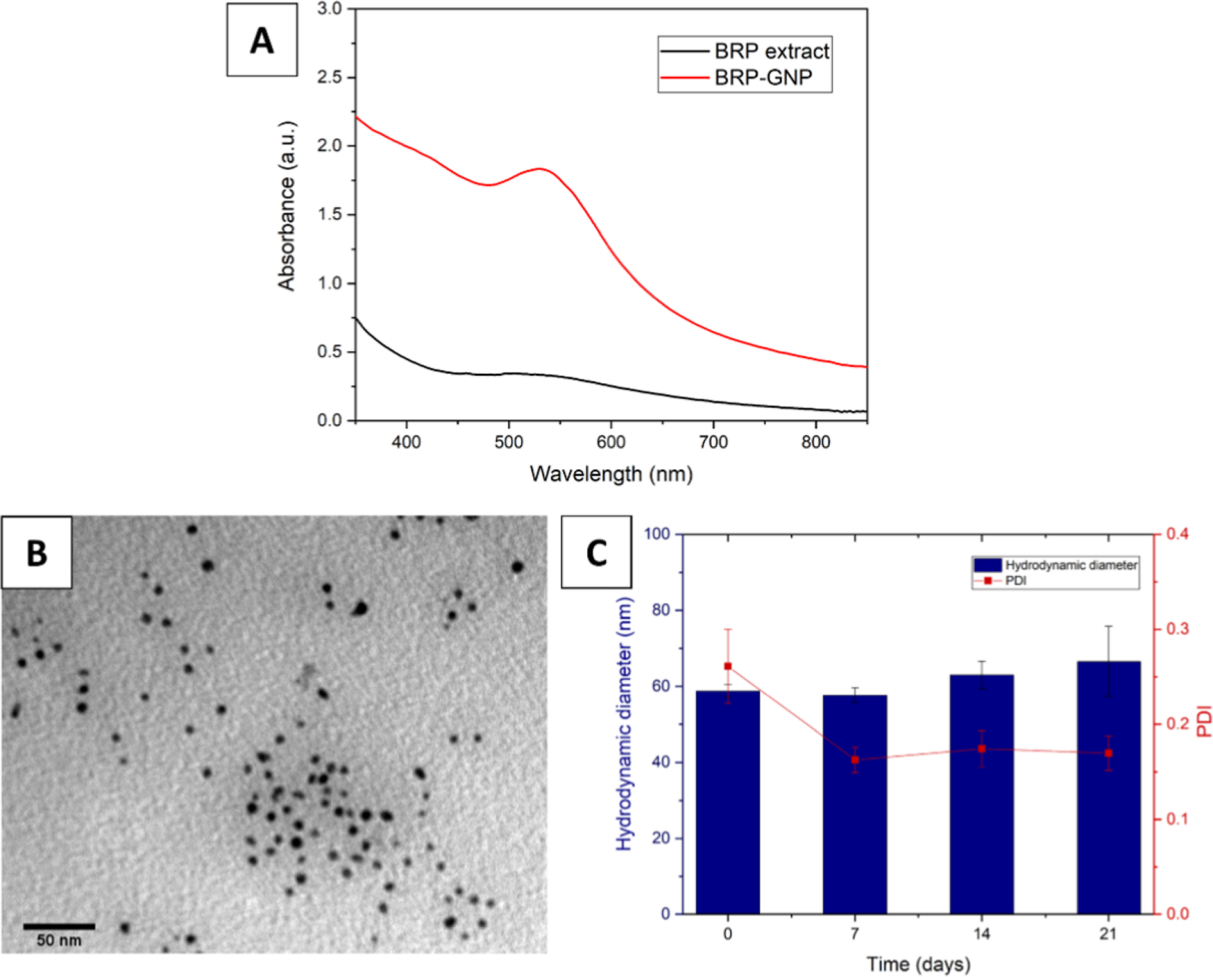
Characterization of BRP-GNPs. (A) UV–vis spectra of BRP extract (black line) and nanoparticles (SPR band) (red line), (B) TEM image acquired in the bright field, and (C) stability over the time (days).

BRP is a natural product made by bees and is found in northeast Brazil. Considered a complex mixture, it is mainly composed of phenolic compounds, such as flavonoids and isoflavonoids (e.g., liquiritigenin, formononetin, vestitol, medicarpin, and biochanin A) and prenylated benzophenones (e.g., oblongifolin B and guttiferone E).^30−32^ Taking advantage of the reduction potential of these molecules and their ability to chelate metals, we used the BRP crude extract to produce GNPs using a green synthesis method.^32^ The formation of the BRP-GNPs was confirmed by the UV–visible (UV–vis) spectrum that demonstrated the appearance of a surface plasmon resonance (SPR) band at 523 nm (Figure 1A), a phenomenon that occurs on the surface of metallic nanoparticles (gold and silver, for example) due to the coherent oscillation of free electrons.^33,34^ GNPs mostly spherical and quasi-spherical shaped with an average size of 6.34 ± 2.01 nm measured by transmission electron microscopy (TEM) were obtained (Figure 1B). The hydrodynamic diameter was equal to 58.79 ± 1.69 nm, and the polydispersity index (PDI) was 0.26 ± 0.04. The formulation was stable for 21 days, according to the analysis of variance ANOVA (Tukey’s test, *p* < 0.05) (Figure 1C). The zeta potential value was negative (ZP = −17.20 mV).

**Figure 2:**
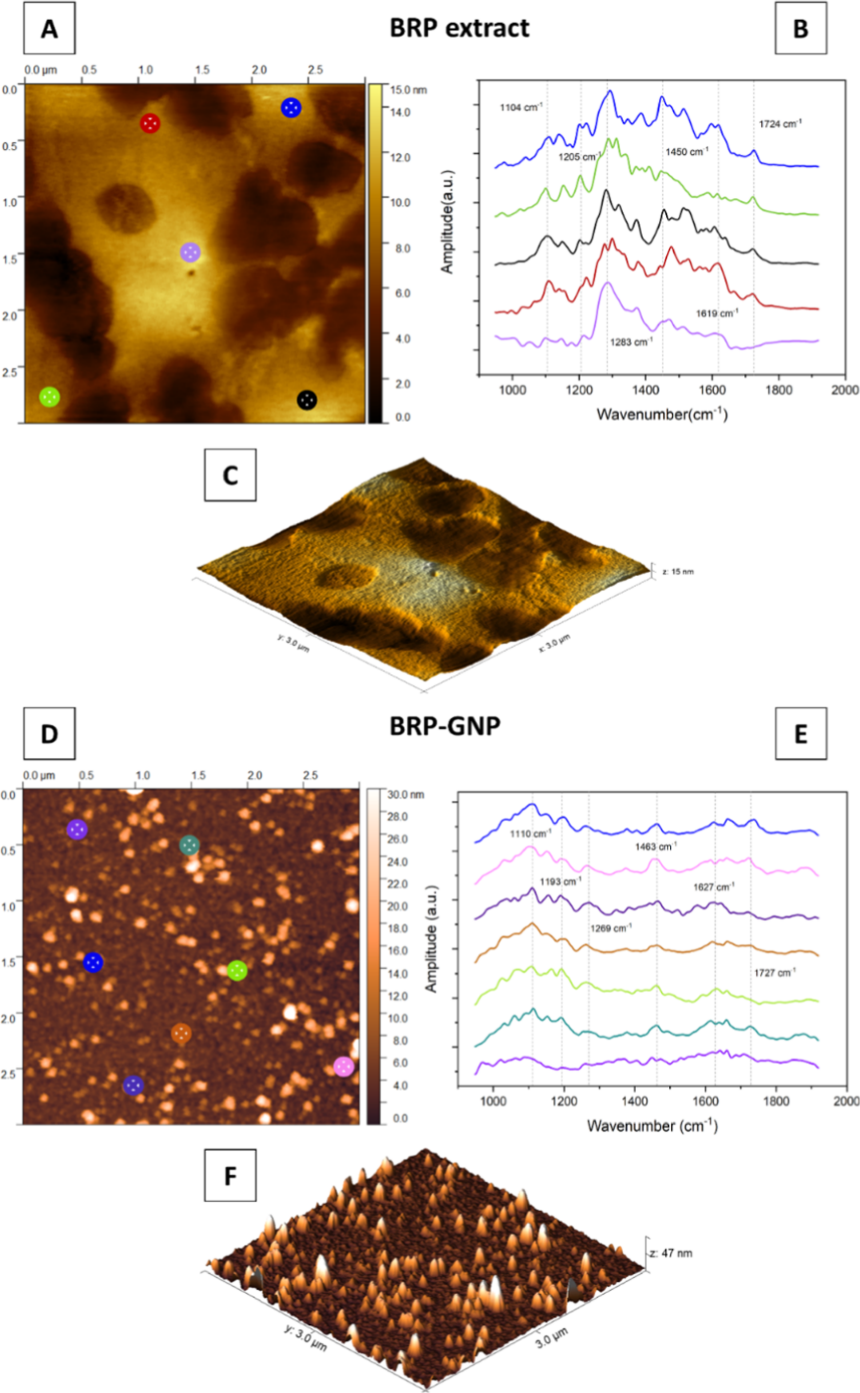
AFM-IR characterizations of BRP extract and BRP-GNPs. AFM images showing the regions where IR spectra were collected on samples of (A) BRP extract and (D) BRP-GNPs. IR spectra collected at various points of the sample: (B) BRP extract and (E) BRP-GNPs. Topographical images of (C) BRP extract and (F) BRP-GNPs.

To understand the propolis compounds capping around GNPs, we evaluated BRP extract and BRP-GNPs by atomic force microscopy coupled with the infrared spectroscopy (AFM-IR) technique. Figure 2A,C shows AFM measurements to the topographic profile of the BRP extract and the IR spectra collected from different regions of the sample (Figure 2B). The 2000–1600 cm^–1^ range can be related to the C–H bond in aromatic compounds.^35^ Absorption peaks at 1619 and 1450 cm^–1^ are related to the stretching in C=C bonds in aromatic rings observed in phenolic compounds.^36,37^ The bands in the 1000–1110 cm^–1^ range can be attributed to the C–O stretching ester group and/or to secondary alcohols observed in flavonoids.^38,39^ In addition, the 1283 cm^–1^ band refers to C–O in polyols, such as hydroxyflavonoids.^40^ Stretching of carbonyl aliphatic ketone (C=O) at 1724 cm^–1^ was also observed.^41,42^

The difference between the AFM images of the BRP extract (Figure 2A,C) and those of the BRP-GNPs (Figure 2D,F) is clear. In the former, no defined forms are identified, while in the latter, the presence of nanoparticles was visible. BRP-GNPs demonstrated an average size of 7.19 ± 4.53 nm (*n* = 100) measured by AFM, consistent with TEM measurements. Furthermore, Figure 2E confirms that the functional groups of BRP are covering GNPs’ surface. The IR spectra revealed absorption peaks similar to those of BRP extract, including 1463 and 1627 cm^–1^ (C=O bonds) and 1269 cm^–1^ (C–O groups in phenols). The slight shifts observed in the absorption peak values may be due to interactions between the functional groups and the surface of the nanoparticles.^43^ These results suggest the involvement of phytochemicals in the reduction of Au metal ions to GNPs and also indicate their role as stabilizing agents.^44^ Biogenic nanoparticles functionalized with natural compounds from medicinal plants are frequently described in the literature as exhibiting interesting biological activities such as antimicrobial, anti-inflammatory, antioxidant, and anticancer activities.^45^ We have found that BRP-GNPs exhibit antimicrobial activities against several types of microorganisms,^32^ which enables their application in the field of agriculture.

### 2.2 Phytotoxicity Assay

Currently, there are no specific tests for evaluation of the phytotoxicity of nanomaterials.^46,47^ The U.S. EPA (United States Environmental Protection Agency) and OECD guidelines for chemical tests are generally employed for plant nanotoxicity assays.^48^ Therefore, there is a growing need to develop methodologies tailored to evaluate the phytotoxicity of nanomaterials considering their distinct physicochemical properties and modes of interaction with plants. In this study, the seeds were treated with free BRP extract and BRP-GNPs at different concentrations, and the effects on germination rate and seedling length were verified. According to the results, free BRP extract and green synthesized GNPs showed different phytotoxicity after seed treatments, depending on the plant species and method employed.

The dry weight of the seedlings after treatment with BRP extract showed a loss of biomass of 3.96% (wheat), 4.97% (soybean), and 2.31% (tomato) compared to the control. In contrast, treatment with BRP-GNPs resulted in a more significant decrease in dry weight for wheat (11.47%) and tomato seedlings (8.80%), while the decrease was less pronounced for soybean (2.61%) (Table 1). The effects of nanoparticles on seed germination are influenced by several factors, including their size, shape, surface charge, type of capping agents, and concentration.^49^ In this study, we observed that the dry biomass of wheat and tomato seedlings decreased significantly after exposure to BRP-GNPs. This reduction may be attributed to exposure to BRP-GNPs, which could induce oxidative stress, cellular damage, or alterations in the hormonal balance in the plants. However, further research is needed to fully elucidate the specific effects of BRP-GNPs on seed development and growth.^50,51^

**Table 1:** Dry Weight of Seedlings Treated with Brazilian Red Propolis (BRP Extract) and GNPs Synthesized with BRP (BRP-GNPs)^a^

	dry weight (mg)		
	wheat	soybean	tomato
control	28.49 ± 6.43	149.63 ± 17.42	2.16 ± 0.26
BRP extract	27.36 ± 5.74	142.20 ± 21.54	2.11 ± 0.55
BRP-GNP	25.22 ± 6.90	145.72 ± 19.17	1.97 ± 0.45

a Control: seed treated with water.

**Figure 3:**
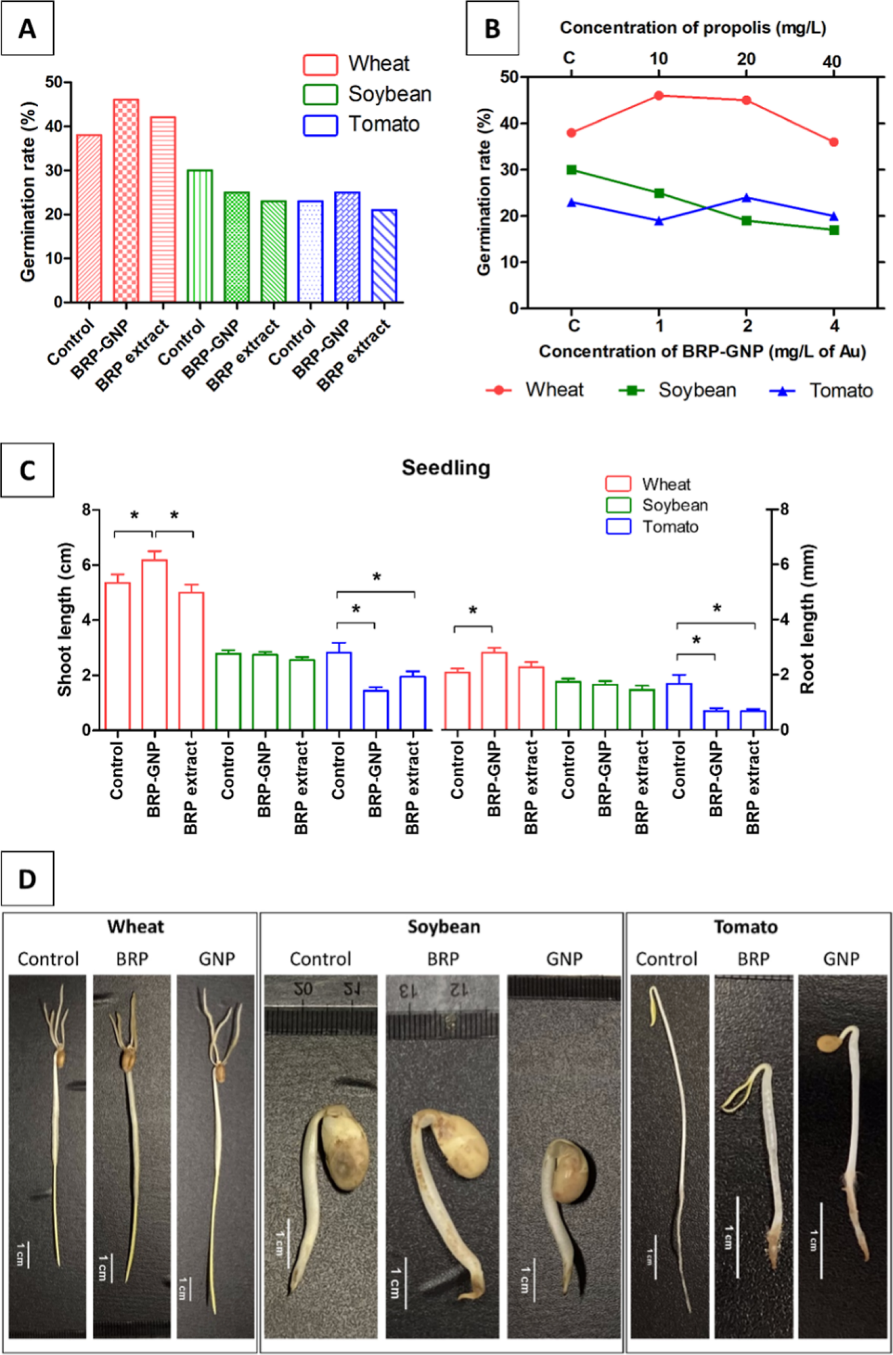
Effects of BRP extract and BRP-GNPs on seed germination and seedling growth. (A) Percentage of germination of seeds (wheat, soybean, and tomato) treated with BRP extract, BRP-GNPs, and control groups (treated with water). The concentration used in both treatment groups was equal to 10 mg/L of propolis. The germination index was evaluated after 5 days; the number of seeds used in each treatment was equal to 50 (*n* = 50). (B) Percentage of germination after seed treatment with BRP-GNPs at different concentrations. (C) Measurements in shoot length and root of seedlings. The (*) symbols on the graph represent seedling length values significantly (*p* < 0.05) different from those in the control group. (D) Seedling (wheat, soybean, and tomato) treated with BRP extract, BRP-GNPs, and control groups (treated with water) after 5 days of sowing.

Despite the weight loss of biomass, positive effects were observed after wheat seeds were treated with BRP-GNPs. Interestingly, seedling lengths were significantly higher (Tukey’s test; *p* < 0.05) than in the control group (1.21-fold) and the group treated with the free extract (1.24-fold). Another point observed is that low concentrations (1 and 2 mg/L) of GNPs have positively influenced the germination index, whereas the higher one (4 mg/L) negatively affected it (Figure 3A,B). Moreover, these results suggested that the toxicity of biogenic GNPs is dose-dependent. It has been reported that seed treatment using GNPs helped increase water intake in maize plants.^52^ Water uptake is primordial for seed germination as mature seeds are often dried in nature and request large amounts of water to initiate the metabolism and growth of the cells.^53^ Nonetheless, nanoparticles in high concentration can adhere to the pores of the cell wall of the root system, inhibiting water transport.^54^ Previous studies have found that GNPs at low concentrations demonstrated growth-promoting effects, whereas GNPs at high concentrations (≥100 mg/L) generated detrimental effects on plants.^55,56^ These findings align with our results, indicating that low doses of GNPs positively impact plant growth. Furthermore, treatment of wheat seeds with BRP-GNPs led to more positive and relevant effects on seed germination and seedling growth than treatment with the free BRP extract. These effects could be related to the high surface area-to-volume ratio of nanoparticles that can facilitate easy transportation in the plant system and their interaction with molecules involved in the photo stimulatory actions such as phytohormones.^1,57^ This is supported by the observed germination rate in seeds exposed to BRP-GNPs, which was 21% higher than that of the control group. GNPs can induce structural and functional reorganization of the photosynthetic apparatus in wheat plants, leading to increased photosynthesis rates.^58^ This effect can be attributed to the SPR effect of BRP-GNPs and the propolis compounds on the nanoparticle surface, facilitating energy transfer in the light-harvesting complex (LHC) and thereby improving light absorption and metabolic processes during the early stages of wheat plant development.^58,59^ These results confirm that moderate stressors can stimulate living organisms, while high doses may result in adverse effects.^60^

As shown in Figure 3A, the BRP-GNPs and BRP treatments demonstrated an improved germination rate in wheat seeds. Also, positive effects were maintained in the seedlings, showing an increase in shoot and root elongation compared with the control group (Figure 3C). These positive effects were not observed for soybeans and tomatoes. The differential response of roots and shoots to BRP-GNPs and BRP extract treatments can be attributed to several mechanisms. Root alteration is often one of the primary indicators of nanoparticle toxicity.^61^ Multiple studies have documented that metal nanoparticles, including silver (Ag),^62^ zinc oxide (ZnO),^63^ and iron oxide (FeO and Fe_2_O_3_),^64^ commonly reduce root elongation across various plant species. For instance, ZnO nanoparticles have been shown to significantly reduce the root size of *Lolium perenne* (ryegrass), causing vacuolation and collapse of epidermal and cortical root cells.^63^ Similarly, Deng et al.^65^ observed that copper oxide nanoparticles notably reduced the root structure in *Allium cepa*. In another study, Sun and colleagues (2019)^66^ found that starch-stabilized zerovalent iron nanoparticles accumulated more in the roots than in the shoots of mung bean seedlings, suggesting that the roots were more susceptible due to this differential accumulation. Furthermore, the deposition of insoluble metal ions or nanoparticles in the root zone may hinder nutrient uptake, enhancing the phytotoxicity of the root. Additionally, metal nanoparticles can disrupt cellular integrity, leading to root dysfunction.^66^

No significant alterations were observed in the shoot and root lengths of soybean seedlings exposed to the same free BRP extract and BRP-GNPs concentration compared with the control group (Figure 3C). However, there was a decrease in seed germination at 23.33% and 16.66% compared to the control (Figure 3A) after both treatments (free BRP extract and BRP-GNPs, respectively). As observed before, the lowest concentration caused a less negative or no effect on the germination rate and root elongation, indicating once again that toxic effects begin to be generated from a concentration threshold. Different plant species can exhibit distinct biological responses to the same type of nanoparticles, which may act as either biostimulants or phytotoxic agents. Studies have demonstrated that monocotyledonous (e.g., wheat) and dicotyledonous (e.g., soybean, tomato) plants interact differently with nanoparticles due to variations in absorption pathways, translocation mechanisms, and metabolic responses.^67−69^ Soybean has been a model for studies of toxicity and accumulation of metals due to its high biomass and simplicity of cultivation.^70^ Exposure of soybeans to nanoparticles leads to particle uptake, translocation, biotransformation, and bioaccumulation,^71^ processes that can initiate beneficial or harmful effects on the physiology and anatomy of the soybean plant.^72^ In this study, no benefits of BRP-GNPs on soybeans were observed, with only nonsignificant (Tukey’s test; *p* < 0.05) adverse effects on germination rate and no effects on the seedlings elongation, contrasting with the effects observed in wheat and tomato seeds. This result underscores the importance of examining multiple plant species in studies investigating the impact of nanoparticles on agriculture.

The tomato seeds were treated by the priming method in which the seed underwent prolonged exposure to the sample of interest compared to the coating method. This method has been described in other studies as effective in improving physiological parameters, such as photosynthetic rate, stomatal conductance, transpiration rate, plant height, and dry biomass of tomato plants.^73,74^ Nevertheless, the priming process can potentially affect seeds’ metabolic activity at cellular and molecular levels, leading to positive or toxic effects.^12^ We observed a seed germination rate 16.00% lower for free BRP extract and 8.70% higher for the group exposed to BRP-GNPs than that of the control group (Figure 3A,B). The mechanisms by which nanopriming induces seed germination are unclear.^75^ A few possibilities have been suggested, such as the formation of nanopores in the shoot, which enhances water uptake, activation of ROS/antioxidant cascade in seeds, generation of hydroxyl radicals to loosen the cell wall, and induction of rapid hydrolysis of starch.^52,76^ Nanoparticles stimulate ROS when they enter the seed coat.^77^ Optimum levels of ROS are fundamental to disrupt seed dormancy and provoke germination.^78^ However, elevated ROS can stimulate oxidative stress,^79^ inhibition of photosynthesis, and the exchange of gases, leading to decrease in plant growth and biomass.^80^ Thus, the concentration of nanoparticles used in the nanopriming technique determines the positive or cytotoxic/genotoxic effects. For example, low concentrations of AgNPs have been associated with improvements in germination rate and root length due to low levels of ROS production.^81,82^ Nevertheless, seedling growth was harmed when the seeds were treated with elevated concentrations of AgNPs. Another possibility hypothesized in the literature is that the movement of GNPs within the roots facilitates the transport and distribution of macro- and microelements within the plant, increasing the nutrient availability and growth of root and shoot in tomato seedlings.^52,83^ Although we observed an increase in the germination rate of seeds treated with BRP-GNPs first, decreases in shoot and root lengths were observed after both treatments (BRP extract and BRP-GNPs) (Figure 3C,D). In the priming technique, seeds undergo prolonged exposure to the extract solution and nanoparticles as they remain soaked longer than in the coating method, allowing stressors to penetrate and generate beneficial or toxic effects. Thus, the priming method may impact the penetration of nanoparticles or propolis compounds, thereby exacerbating their effects.

These results highlight that plant species, stage of development, and prefarming methods are factors that influence phytotoxicity.^56,84^ Furthermore, the size, shape, concentration, and chemical surface of nanoparticles affect not only phytotoxicity but also the parameters of seed quality, such as germination percentage, seedling vigor and elongation, and dry weight.^56,85,86^ Amooaghaie et al. (2015)^87^ reported that chemically synthesized AgNPs (30 nm) displayed a higher inhibitory effect on seed germination and seedling growth than green-synthesized AgNPs (15 nm) in six different seedlings (Lolium, wheat, bean, common vetch, canola, and lettuce). Furthermore, chemically synthesized GNPs (15 nm and spherical shape) and phytosynthesized GNPs (10–30 nm and anisotropic morphology) demonstrated different phytotoxicity after maize seed nanopriming. Thus, both the physical–chemical properties of GNPs and the compounds utilized during their synthesis can potentially influence the toxic effects of the nanoparticles.

BRP extract contains a high content of polyphenolic compounds, including flavonoids and isoflavonoids.^30,31^ Flavonoids are components of plant seeds and play significant roles in seed germination,^88^ seed growth, and development.^89^ The functions of flavonoids as growth stimulators and stress mediators have been investigated in many studies.^90−92^ Some flavonoids act as signaling compounds in phytomicrobial associations in the seedlings of legumes.^93^ On the other hand, determined classes of flavonoids secreted from germinating seeds and roots display phytoinhibitory activities, inhibiting seedling growth in a dose-dependent manner. Depending on the class and type, flavonoids may be stimulatory (usually at lower concentrations) or inhibitory (at higher concentrations).^94,95^

In our results, we observed that propolis concentrations up to 10 mg/L stimulated the germination of wheat seeds but did not significantly impact seedling growth compared to the water-treated control. In the soybean experiment, we did not detect any positive or negative impact on the germination rate and growth of seedlings treated with BRP extract concentrations of up to 7.5 mg/L (data not shown). However, we noticed a slight decrease in the germination rate of seeds treated with concentrations above 10 mg/L. These results are consistent with another study on soybeans in which flavonoid treatment did not affect seed germination under ideal growing conditions.^92^

For the tomato experiment, concentrations up to 5 mg/L BRP extract showed a positive impact on seedling growth (data not shown), but higher concentrations negatively affected both seed germination and seedling growth. Similarly, low concentrations of the flavonoid rutin (1 to 5 mM) did not induce any impacts (positive or negative) on tomato seedlings treated by the nanopriming method, while high concentrations (10 and 20 mM) caused negative impacts on growth of the seedlings’ roots.^96^ Briefly, seed germination experiments indicated that the BRP extract and biogenic GNPs at low concentrations did not have any severe effect on seed germination and seedling growth. In contrast, high concentrations negatively affected them. In addition, BRP-GNPs induced positive and relevant effects on the germination and growth of wheat seedlings, showing their potential as a stimulant in wheat crops. The soybean seedling growth assay was the least impacted by the treatments, while the tomato seed experiments were the most negatively affected. Interestingly, biogenic GNPs provided better enhancement of germination than free BRP extract. Overall, our study revealed that the negative effects of phytosynthesized BRP-GNPs on seedlings could be partly attributed to the propolis coating itself since we also observed harmful effects in the groups treated with free BRP extract. These BRP-GNPs have been used for the first time in a germination assay; thus, they need further evaluation using different techniques and more in-depth investigations.

### 2.3 Fish Embryo Acute Toxicity Test

**Figure 4:**
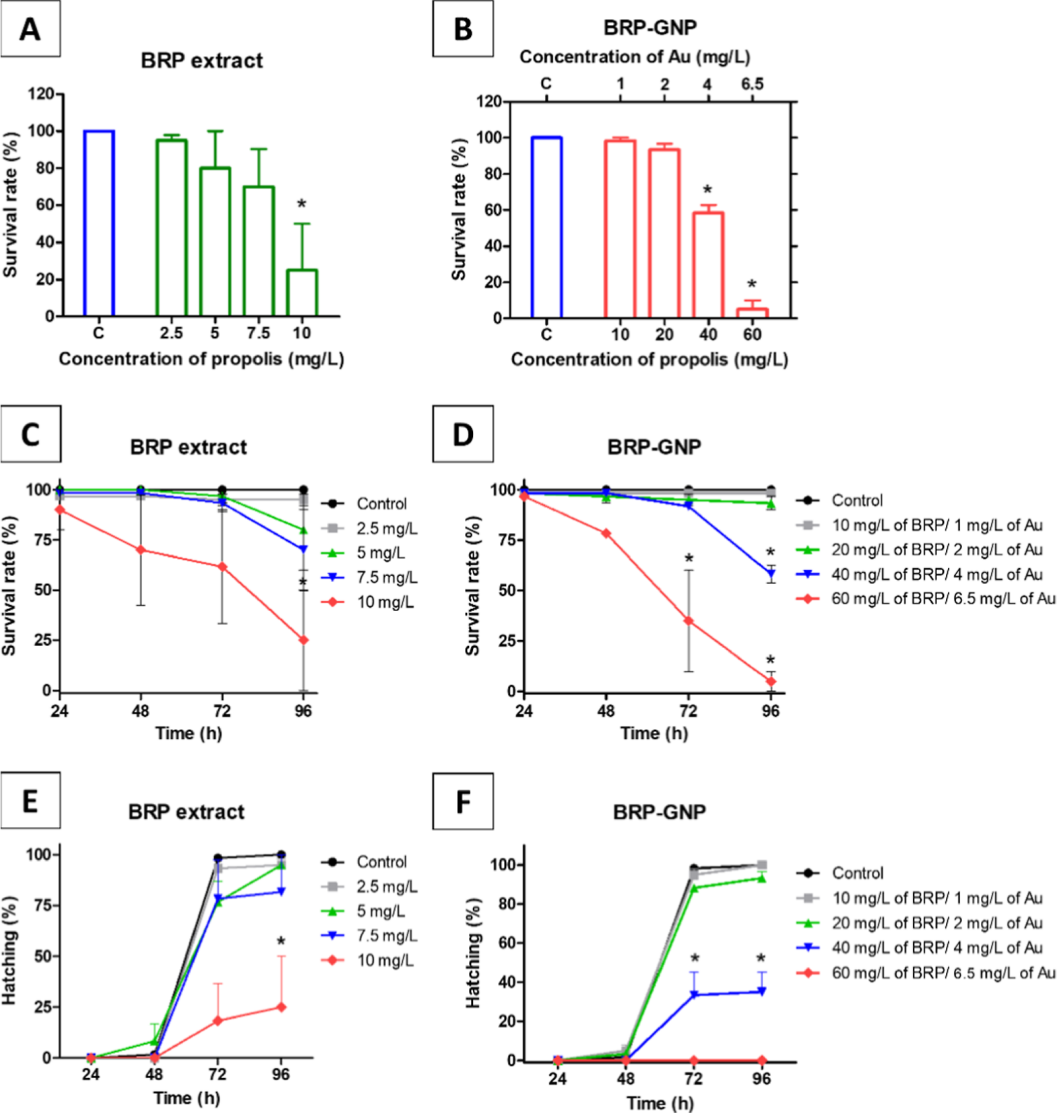
Fish embryo acute toxicity assay. Viability of zebrafish embryos treated with different concentrations of (A) BRP extract and (B) BRP-GNPs. The survival rate of zebrafish embryos after exposure to (C) BRP extract and (D) BRP-GNPs at different concentrations. Embryo hatching rate after time of exposure of (E) BRP extract and (F) BRP-GNPs. The (*) symbols on the graph represent survival rate values significantly (*p* < 0.05) different from the control group (represented by the letter C on the graph).

All animals showed normal development in the control group. Toxic effects were observed in groups treated with the BRP extract and BRP-GNPs at different concentrations. For the group treated with the BRP extract at a concentration of 2.5 mg/L, 95% viability was observed. The group treated with 5 mg/L BRP showed 80% viability, but the larvae did not show swim bladder development, indicating a delay in development. The last BRP extract concentration studied (10 mg/L) induced high toxicity in the embryos, causing the appearance of deformed larvae. The calculated LC_50_ value was 10.67 mg/L (Figure 4A). These results are similar to those obtained by Aldana-Mejia et al. (2021),^97^ who reported an LC_50_ value equivalent to 9.37 mg/L for the BRP extract.

**Figure 5:**
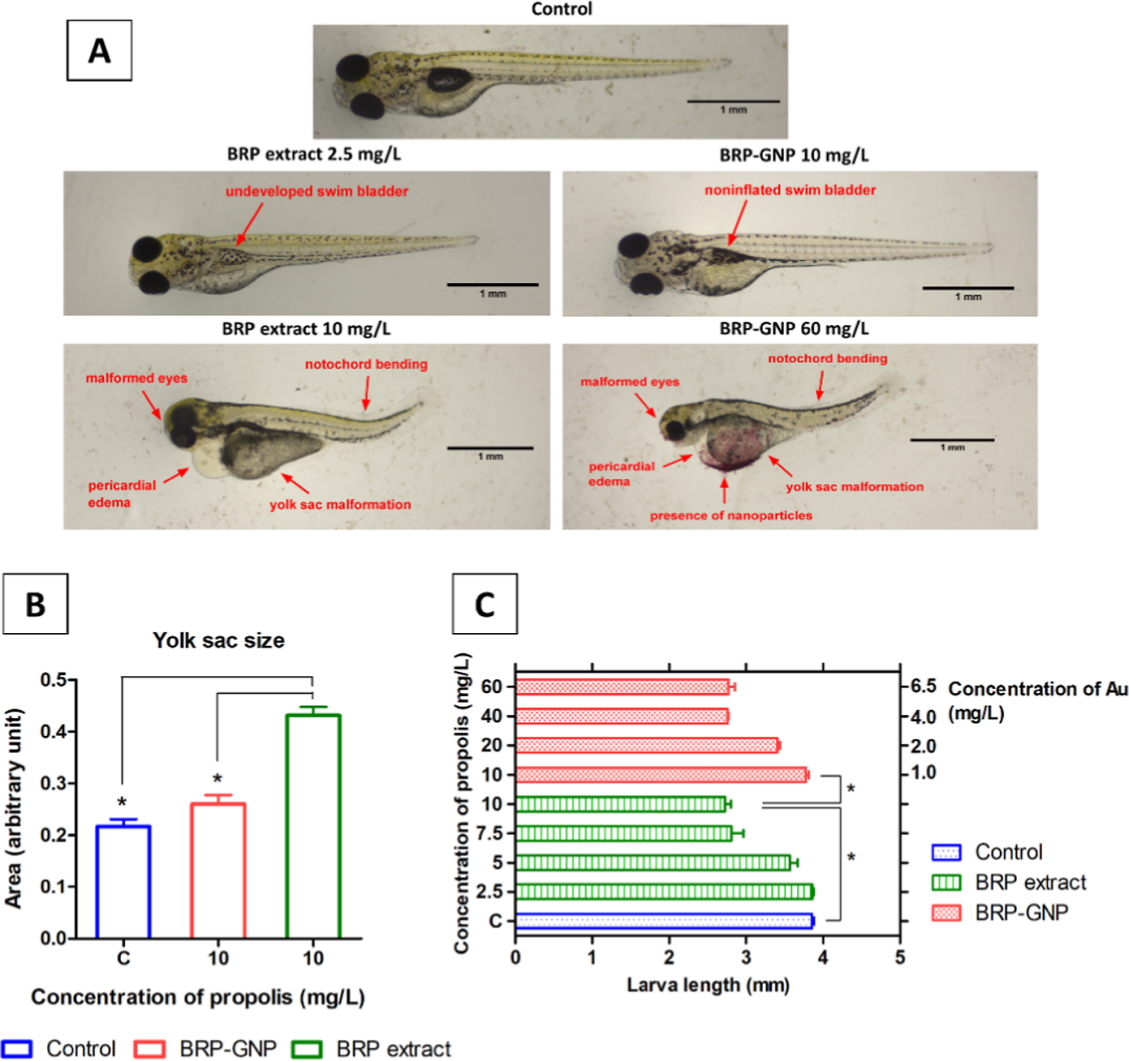
Toxic effects of BRP extract and BRP-GNP on zebrafish embryos. (A) Zebrafish larvae at 96 h after time of exposure to BRP extract and BRP-GNPs. The treatment concentrations are expressed in mg/L of propolis. (B) Size changes in embryos treated with BRP extract and BRP-GNP in the YS. (C) Length of the embryos in relation to the different concentrations of BRP extract and BRP-GNPs. The (*) symbols on the graph represent larva length significantly (*p* < 0.05) different from that in the control group.

BRP-GNP concentrations up to 20 mg/L showed low toxicity and more than 90% viability (Figure 4B,D). However, some larvae did not show an inflated swim bladder (Figure 5A). Concentrations higher than 40 mg/L demonstrated significant toxicity and the presence of larvae with anomalies. The LC_50_ calculated was 40.17 mg/L (equivalent to 4 mg/L of Au concentration). Several studies support a general nontoxic effect of GNPs in zebrafish and other aquatic organisms.^98−100^ However, it is known that the toxicity of nanoparticles varies with different coating agents.^101^ For example, Sadhukhan et al. (2022)^100^ reported that organometallic gold-based folate nanoparticles (FGNPs) did not induce any adverse effects in a brain inflammation model in zebrafish, considering them toxicologically safe. However, Krishnaraj et al. (2022)^102^ found that a concentration of 200 μg/mL of GNPs synthesized from *Angelica keiskei* extract was highly toxic to fish embryos.

Comparing Figure 4C,D, the treatment with the BRP extract was more toxic to the embryos, a fact made evident by the selection of the concentration range for this study. While the highest concentration of the free extract used (10 mg/L) was the most toxic to the embryos, showing a reduction in viability of more than 60%, the same concentration of BRP-GNPs (equivalent to 10 mg/L BRP extract and 1 mg/L Au concentration) was considered to be of low toxicity, showing a viability greater than 90%. In our latest work,^32^ we verified that BRP-GNPs were less cytotoxic to cancer cells than free BRP extract, suggesting a loss of activity of phytochemicals present in the extract. Similar results were observed by Machado et al. (2021)^103^ with GNPs prepared using two species of algae *Cystoseira tamariscifolia* (CT) and *Cystoseira baccata* (CB). During the process of green synthesis of metallic nanoparticles, oxidation of some active compounds in plant extracts occurs to reduce metal ions.^104^ Due to the oxidation process, after the preparation of the nanoparticles, the oxidized molecules bound to the surface of the particles may present a loss of their biological activities depending on the degree of involvement of each compound during the oxidation–reduction reaction.^32^ Phenolic compounds have been indicated as the main reducing and capping agents involved in the synthesis of metallic nanoparticles.^105^ As previously discussed, the main functional groups of phytochemical compounds from the propolis extract on the surface of BRP-GNPs were identified using AFM-IR, suggesting the capping of propolis compounds on the surface of GNPs. Considering the results of the physicochemical characterization and toxicity evaluation (survival rate (%) of embryos), these findings indicate that the biological properties of BRP-GNPs can be attributed to the remaining activity of the BRP present on the GNP’s surface. Compared with the control group, the treatments in general interfered with the normal development of zebrafish embryos.

Malformations in the groups treated with BRP extract and BRP-GNPs increased dose-dependently (Figure 5A). The significant anomalies observed at 96 h, at the highest concentration of each treated group, included bending in the spine, notochord, and tail, pericardial edema, eye dysplasia, yolk sac (YS) edema, slow heart rate, incomplete swim bladder development, and decrease in length of larvae when compared to the control group (Figure 5A,C). Furthermore, some larvae exhibited behavioral disturbances such as agitation, erratic swimming, and difficulty floating. These effects can be related to some chemical markers of BRP, mainly from the flavonoid class, which can affect the central nervous system, leading to complex effects on early life fish neurodevelopment and neurobehavioral alterations.^97,106^ The toxicity of flavonoids including formononetin and biochanin A, flavonoids present in BRP extract, in fish larval embryos has been described in the literature.^97,106^ Furthermore, the hydroethanolic extract of *Dalbergia ecastaphyllum*, the primary botanical source of flavonoids from red propolis,^107^ demonstrated acute toxicity in adult fish, resulting in high lethality after exposure to extract concentrations ranging from 25 to 100 mg/L. Additionally, individuals exhibited behavioral disturbances and signs of intoxication.^108^

The hatching rate of the control group in 72 h was 98.33%, while those in the groups treated with BRP-GNPs (Figure 4F) at concentrations of 10, 20, 40, and 60 mg/L (equivalent to 1, 2, 4, and 6.5 mg/L of Au concentration, respectively) were 95.00%, 88.33%, 33.33%, and 10.00%, respectively, with the last two concentrations showing significantly lower hatching rates than the control group (*p* < 0.05). The hatching rates in the groups treated with BRP extract (Figure 4E) were 93.33%, 60.00%, 78.33%, and 38.33% when the concentrations were 2.5, 5, 7.5, and 10 mg/L, respectively. The highest concentration generated a hatching rate significantly lower than that of the control group (Figure 4E). Some of these toxic effects may be related to the structure of the embryos. Chorion is a biological barrier that involves the development of an embryo until its hatching.^109^ This special structure has pore channels with diameters of up to 500 nm^110^ that allow gas supply and impede some pollutants from passing through.^109^ It is considered that nanoparticles with small sizes penetrate the chorion.^111^ In contrast, macromolecules, such as the BRP extract or nanoparticles with larger sizes that have high affinity to the embryonic chorions, cannot penetrate the chorion but can adhere to the chorionic surface and cause toxicological effects.^112^ The embryonic development stage is crucial in the life cycle of a fish. Any impairment affecting embryo functions can lead to embryo deformity, hypoxia, and developmental disorders.^113^ Knowing that BRP extract is a complex mixture composed of waxes and other lipophilic molecules, we suggest that some molecules may have interacted or adhered to the surface of the chorion, impairing the supply of gases and partially contributing to the toxic effects in the embryo. The increase in toxicity at the highest concentrations of BRP extract (10 mg/L) due to the interaction with the chorion, or even due to excess of GNPs (60 mg/L BRP concentration and 6.5 mg/L Au concentration), can explain the decrease in the hatching rate observed between 48 and 72 h, resulting from the death of embryos in this concentration range.

Size measurements from the control group showed that larvae were 3.86 ± 0.05 mm in length. At concentrations of 10 mg/L of free BRP extract and BRP-GNPs, larvae were found measuring 2.73 ± 0.13 and 3.77 ± 0.07 mm in length, respectively. These results indicated that BRP extract treatment led to about a 30% decrease in the size of larvae when compared with both control and BRP-GNP-treated groups (Figure 5C). Another factor studied was the increase in the area of the YS after the treatments. Compared to the control, treatment with free BRP extract (10 mg/L) induced an approximately 2-fold increase in the YS area (Figure 5B). The increase in the YS may be related to the yolk retention due to decreased yolk utilization or to the presence of edema (fluid accumulation within the YS) resulting from an impaired osmotic gradient regulation, leading to excessive water uptake into the embryo.^114,115^ Lipophilic xenobiotics, such as organic compounds in BRP extract, are known to accumulate in the YS and may lead to its enlargement.^116,117^ These compounds can be internalized by the YS epithelium through passive and active transportation mechanisms, including diffusion, receptor-mediated endocytosis, or pinocytosis.^114,118^ Halback and co-workers (2020)^119^ showed that 95% 4-iodophenol and 67% carbamazepine, two lipophilic substances, were found in the YS in 26 hpf (hours post fertilization) embryos. The authors suggested that the concentration distribution of the compounds within zebrafish embryos may not follow a simple partitioning between the embryo and the exposure solution but could be influenced by metabolic biotransformation and active transport. YS edema is a well-documented toxicological response in zebrafish developmental toxicity studies involving plant extracts.^97,120,121^

No significant changes were observed for the same concentration (10 mg/L) of the BRP-GNPs. However, from the concentration of 60 mg/L BRP-GNP (equivalent to 6.5 mg/L Au concentration), not only a significant increase in the size of the YS but also the presence of nanoparticles in this region was observed. The increase in YS size after exposure to nanoparticles has also been observed by other authors.^111,122^ The high lipid content of the YS may be a target for nanoparticle accumulation and lipophilic molecules and may influence the distribution of nanoparticles all over the body through lipid migration during nutrient consumption by the embryo.^111,116^

In fish eggs, the endogenous lipid reserves are accumulated in the form of yolk^123^ and are utilized by the embryo during development.^124^ Any damage to the YS or the accumulation of nanoparticles and organic compounds in this region may affect the supply of nutrients to the embryo. The consequence is insufficient nutrient metabolism, which could lead to abnormal embryonic development,^125^ as observed in the results of BRP extract and BRP-GNP treatments. The increase in the YS by the accumulation of nanoparticles or BRP extract compounds may also be related to the appearance of smaller larvae, as previously demonstrated (Figure 5B), since the suppression of nutrient consumption from the yolk can lead to delayed development of the organs, resulting in a short body length.^126,127^ It has been reported that exposing embryos to high concentrations of isoliquiritigenin, a flavonoid commonly found in red propolis, led to YS retention and decreased larvae body length.^127^

On the other hand, we observed that the YS size of the control group started to decrease between 72 hpf (hour postfertilization) and 96 hpf after hatching, suggesting that the YS was being utilized during the development.^125^ These results have proven that the toxicity of BRP-GNPs observed in the present study may be due to the presence of BRP extract on the nanoparticles’ surface.

Overall, these results highlight the potential of BRP-GNPs in promoting seed germination and seedling growth, suggesting its promising application in enhancing crop productivity and stress tolerance. However, negative effects were observed in the seed and in the zebrafish embryos, indicating the potential problems of use and/or discard of this nanostructure in ecological systems. Although no severe impacts were observed following exposure of species with low doses of BRP-GNPs, in the future, this may be a concern as concentrations of GNPs are expected to increase in the environment. Due to their small size (6.34 ± 2.01 nm), BRP-GNPs could pose risks to humans and wildlife by interacting with tissues, cells, and body fluids through various pathways. Studies have shown that nanoparticles can reach the central nervous system and may lead to inflammation in the lungs, liver, and spleen through mechanisms involving cellular uptake and oxidative stress induction.^28,128^ Inorganic nanoparticles, including BRP-GNPs, can bioaccumulate depending on their size, shape, and surface properties, potentially resulting in tissue damage over time.^27,29^ Further research is needed to fully understand the mechanisms underlying these effects and to optimize nanoparticle concentrations for maximum benefit while minimizing potential adverse impacts on plant growth and development as well as on the environment.

## 3 Materials and Methods

### 3.1 Synthesis and Characterization of BRP-GNPs

GNPs were synthesized using BRP extract according to the method developed by Botteon et al. (2021).^32^ Ten milliliters of aqueous solution of 0.50 mM HAuCl_4_·3H_2_O was mixed with 20 μL of BRP extract at mild temperature. The pH was adjusted to 7 using sodium hydroxide (NaOH). The reduction of Au ions was confirmed by the appearance of an absorbance band with a maximum peak at 523 nm by UV–vis spectroscopy (Shimadzu, Kyoto, JPN). Size and morphology of nanoparticles were assessed by TEM using a JEOL model 1200EX instrument (JEOL, Tokyo, JPN) operated at an accelerating voltage at 200 kV. The morphology of the GNP and the chemical characterization of its surface was investigated by an atomic force microscope coupled with an infrared laser, NanoIR2-s (Bruker, Billerica, Massachusetts, USA). Additionally, the average size, PDI, and zeta potential of GNPs were obtained by dynamic light scattering (DLS) analysis (Zetasizer Nano ZS, Malvern, UK).

### 3.2 Phytotoxicity Assay

Wheat (*Triticum aestivum* L), soybean (*Glycine max*), and tomato (*Solanum lycopersicum*) seeds were used in this experiment. For this, 50 healthy and intact seeds were chosen from each plant.^129^ Wheat and soybean seeds were treated with BRP extract (10 mg/L) and 1, 2, and 4 mg/L BRP-GNPs (equivalent to 10, 20, and 40 mg/L in the amount of the BRP extract, respectively) by the coating method and left to dry at room temperature for 2 h. After that, the seeds were sown on germination paper soaked with water and then placed in a germination chamber at 26 °C and left for 5 days. At the end of this time, the seedlings were examined, and the following parameters were evaluated: germination percentage, root elongation, seedling vigor, and dry weight. Tomato seeds were treated by the priming method, submerged in a propolis solution and in dispersion with nanoparticles, and left stirring for 4 h. Afterward, they were placed to dry at room temperature for 24 h. The sowing stage and the analyzed parameters followed the same protocol as described above.

### 3.3 Fish Embryo Acute Toxicity Test

This study was carried out to determine the acute toxicity of BRP extract and BRP-GNPs in zebrafish (*Danio rerio*) embryonic stages. The test was performed as described in (Organisation for Economic Co-operation and Development) OECD guide number 236.^130^ For this test, zebrafish eggs (embryos) were produced by spawning groups in which a breeding group with males and females is placed in spawning tanks a few hours before the onset of darkness on the day before the test. Mating, spawning, and fertilization occurred within 30 min of light onset. After this time, the eggs were carefully collected and washed with distilled water. Embryos considered viable were separated and distributed in 24-well plates (1 egg per well), totaling 20 embryos for each tested sample concentration and 4 control embryos. The wells were filled with 2 mL of the sample at the following concentrations: 2.5, 5, 7.5, and 10 mg/L of free BRP extract and 1, 2, 4, and 6.5 mg/L of BRP-GNPs (equivalent to 10, 20, 40, and 60 mg/L in the amount of the BRP extract, respectively) diluted in medium so that the amount of the sample did not exceed 10% of the total volume of the medium. All concentrations tested were performed in triplicate. The experiment was carried out over a period of time of 24 to 96 h. At the end of 96 h, the plates were taken to the microscope again for the acquisition of the images. The pictures were processed using ImageJ software, and the LC_50_ (lethal concentration for 50% of the exposed embryos) was calculated using the GraphPad Prism 5.0 software.

### 3.4 Statistical Data Analysis

The experimental data were analyzed by using one-way analysis of variance (ANOVA), and the results were represented as mean ± SD (standard deviation). Data were examined using GraphPad Prism, version 5. The significant levels of difference for the evaluated characteristics were calculated, and each of the experimental values was compared to a control group by Tukey’s test at a 95% level. *P* values smaller than or equal to 0.05 were considered statistically significant. The significance level is indicated by an asterisk in the graphs.

## Abbreviations

BRP: Brazilian red propolis
GNPs: gold nanoparticles
SPR: surface plasmon resonance
PDI: polydispersity index
AFM-IR: atomic force microscopy coupled with infrared spectroscopy
hpf: hour postfertilization
YS: yolk sac
FET: fish embryo acute toxicity test

## References

[ref1] Raliya R.; Nair R.; Chavalmane S.; Wang W. N.; Biswas P. Mechanistic Evaluation of Translocation and Physiological Impact of Titanium Dioxide and Zinc Oxide Nanoparticles on the Tomato (Solanum Lycopersicum L.) Plant. Metallomics 2015, 7 (12), 1584–1594. 10.1039/C5MT00168D. 26463441

[ref2] Wohlmuth J.; Tekielska D.; Čechová J.; Baránek M. Interaction of the Nanoparticles and Plants in Selective Growth Stages—Usual Effects and Resulting Impact on Usage Perspectives. Plants 2022, 11 (18), 2405 10.3390/plants11182405. 36145807 PMC9502563

[ref3] Tondey M.; Kalia A.; Singh A.; Dheri G. S.; Taggar M. S.; Nepovimova E.; Krejcar O.; Kuca K. Seed Priming and Coating by Nano-Scale Zinc Oxide Particles Improved Vegetative Growth, Yield and Quality of Fodder Maize (Zea Mays). Agronomy 2021, 11 (4), 729 10.3390/agronomy11040729.

[ref4] Rehman H.; Aziz T.; Farooq M.; Wakeel A.; Rengel Z. Zinc Nutrition in Rice Production Systems: A Review. Plant Soil 2012, 361 (1–2), 203–226. 10.1007/s11104-012-1346-9.

[ref5] Gade A.; Ingle P.; Nimbalkar U.; Rai M.; Raut R.; Vedpathak M.; Jagtap P.; Abd-Elsalam K. A. Nanofertilizers: The Next Generation of Agrochemicals for Long-Term Impact on Sustainability in Farming Systems. Agrochemicals 2023, 2 (2), 257–278. 10.3390/agrochemicals2020017.

[ref6] Balalakshmi C.; Gopinath K.; Govindarajan M.; Lokesh R.; Arumugam A.; Alharbi N. S.; Kadaikunnan S.; Khaled J. M.; Benelli G. Green Synthesis of Gold Nanoparticles Using a Cheap Sphaeranthus Indicus Extract: Impact on Plant Cells and the Aquatic Crustacean Artemia Nauplii. J. Photochem. Photobiol. B Biol. 2017, 173, 598–605. 10.1016/j.jphotobiol.2017.06.040. 28697477

[ref7] Ramalingam V. Multifunctionality of Gold Nanoparticles: Plausible and Convincing Properties. Adv. Colloid Interface Sci. 2019, 271, 101989 10.1016/j.cis.2019.101989. 31330396

[ref8] Kulabhusan P. K.; Tripathi A.; Kant K. Gold Nanoparticles and Plant Pathogens: An Overview and Prospective for Biosensing in Forestry. Sensors 2022, 22 (3), 1259 10.3390/s22031259. 35162004 PMC8840466

[ref9] Sathishkumar M.; Pavagadhi S.; Mahadevan A.; Balasubramanian R. Biosynthesis of Gold Nanoparticles and Related Cytotoxicity Evaluation Using A549 Cells. Ecotoxicol. Environ. Saf. 2015, 114, 232–240. 10.1016/j.ecoenv.2014.03.020. 24835429

[ref10] Saravanan A.; Kumar P. S.; Karishma S.; Vo D. V. N.; Jeevanantham S.; Yaashikaa P. R.; George C. S. A Review on Biosynthesis of Metal Nanoparticles and Its Environmental Applications. Chemosphere 2021, 264, 128580 10.1016/j.chemosphere.2020.128580. 33059285

[ref11] Parveen A.; Mazhari B. B. Z.; Rao S. Impact of Bio-Nanogold on Seed Germination and Seedling Growth in Pennisetum Glaucum. Enzyme Microb. Technol. 2016, 95, 107–111. 10.1016/j.enzmictec.2016.04.005. 27866604

[ref12] do Espirito Santo Pereira A.; Oliveira H. C.; Fraceto L. F.; Santaella C. Nanotechnology Potential in Seed Priming for Sustainable Agriculture. Nanomaterials 2021, 11 (2), 267 10.3390/nano11020267. 33498531 PMC7909549

[ref13] Husen A.; Siddiqi K. S. Phytosynthesis of Nanoparticles: Concept, Controversy and Application. Nanoscale Res. Lett. 2014, 9 (1), 1–24. 10.1186/1556-276X-9-229. 24910577 PMC4031915

[ref14] Malejko J.; Godlewska-Żyłkiewicz B.; Vanek T.; Landa P.; Nath J.; Dror I.; Berkowitz B. Uptake, Translocation, Weathering and Speciation of Gold Nanoparticles in Potato, Radish, Carrot and Lettuce Crops. J. Hazard. Mater. 2021, 418, 126219 10.1016/j.jhazmat.2021.126219. 34102370

[ref15] Farré M.; Gajda-Schrantz K.; Kantiani L.; Barceló D. Ecotoxicity and Analysis of Nanomaterials in the Aquatic Environment. Anal. Bioanal. Chem. 2009, 393 (1), 81–95. 10.1007/s00216-008-2458-1. 18987850

[ref16] Luo P.; Ma G.; Dudkiewicz A.; Mao Z.; Wang L.; Jiang J. Effect of Size and Surface Chemistry of Gold Nanoparticles on Their Retention in a Sediment-Water System and Lumbriculus Variegatus. J. Environ. Sci. Heal. A Tox. Hazard. Subst. Environ. Eng. 2021, 56 (12), 1347–1355. 10.1080/10934529.2021.1996183. 34709127

[ref17] Vaseashta A.; Vaclavikova M.; Vaseashta S.; Gallios G.; Roy P.; Pummakarnchana O. Nanostructures in Environmental Pollution Detection, Monitoring, and Remediation. Sci. Technol. Adv. Mater. 2007, 8 (1–2), 47–59. 10.1016/j.stam.2006.11.003.

[ref18] Zhang Y.; Goss G. G. Nanotechnology in Agriculture: Comparison of the Toxicity between Conventional and Nano-Based Agrochemicals on Non-Target Aquatic Species. J. Hazard. Mater. 2022, 439, 129559 10.1016/j.jhazmat.2022.129559. 35863222

[ref19] Iavicoli I.; Leso V.; Beezhold D. H.; Shvedova A. A. Nanotechnology in Agriculture: Opportunities, Toxicological Implications, and Occupational Risks. Toxicol. Appl. Pharmacol. 2017, 329, 96–111. 10.1016/j.taap.2017.05.025. 28554660 PMC6380358

[ref20] Unrine J. M.; Shoults-Wilson W. A.; Zhurbich O.; Bertsch P. M.; Tsyusko O. V. Trophic Transfer of Au Nanoparticles from Soil along a Simulated Terrestrial Food Chain. Environ. Sci. Technol. 2012, 46 (17), 9753–9760. 10.1021/es3025325. 22897478

[ref21] Judy J. D.; Unrine J. M.; Bertsch P. M. Evidence for Biomagnification of Gold Nanoparticles within a Terrestrial Food Chain. Environ. Sci. Technol. 2011, 45 (2), 776–781. 10.1021/es103031a. 21128683

[ref22] Kamaraj C.; Karthi S.; Reegan A. D.; Balasubramani G.; Ramkumar G.; Kalaivani K.; Zahir A. A.; Deepak P.; Senthil-Nathan S.; Rahman M. M.; Md Towfiqul Islam A. R.; Malafaia G. Green Synthesis of Gold Nanoparticles Using Gracilaria Crassa Leaf Extract and Their Ecotoxicological Potential: Issues to Be Considered. Environ. Res. 2022, 213, 113711 10.1016/j.envres.2022.113711. 35728640

[ref23] Gopinath V.; Priyadarshini S.; Loke M. F.; Arunkumar J.; Marsili E.; MubarakAli D.; Velusamy P.; Vadivelu J. Biogenic Synthesis, Characterization of Antibacterial Silver Nanoparticles and Its Cell Cytotoxicity. Arab. J. Chem. 2017, 10 (8), 1107–1117. 10.1016/j.arabjc.2015.11.011.

[ref24] Ribeiro A. P. C.; Anbu S.; Alegria E. C. B. A.; Fernandes A. R.; Baptista P. V.; Mendes R.; Matias A. S.; Mendes M.; Guedes da Silva M. F. C.; Pombeiro A. J. L. Evaluation of Cell Toxicity and DNA and Protein Binding of Green Synthesized Silver Nanoparticles. Biomed. Pharmacother. 2018, 101, 137–144. 10.1016/j.biopha.2018.02.069. 29482059

[ref25] Rana A.; Yadav K.; Jagadevan S. A Comprehensive Review on Green Synthesis of Nature-Inspired Metal Nanoparticles: Mechanism, Application and Toxicity. J. Clean. Prod. 2020, 272, 122880 10.1016/j.jclepro.2020.122880.

[ref26] Yokel R. A.; MacPhail R. C. Engineered Nanomaterials: Exposures, Hazards, and Risk Prevention. J. Occup. Med. Toxicol. 2011, 6 (1), 7 10.1186/1745-6673-6-7. 21418643 PMC3071337

[ref27] Tran T. K.; Nguyen M. K.; Lin C.; Hoang T. D.; Nguyen T. C.; Lone A. M.; Khedulkar A. P.; Gaballah M. S.; Singh J.; Chung W. J.; Nguyen D. D. Review on Fate, Transport, Toxicity and Health Risk of Nanoparticles in Natural Ecosystems: Emerging Challenges in the Modern Age and Solutions toward a Sustainable Environment. Sci. Total Environ. 2024, 912, 169331 10.1016/j.scitotenv.2023.169331. 38103619

[ref28] Kumah E. A.; Fopa R. D.; Harati S.; Boadu P.; Zohoori F. V.; Pak T. Human and Environmental Impacts of Nanoparticles: A Scoping Review of the Current Literature. BMC Public Health 2023, 23 (1), 1059 10.1186/s12889-023-15958-4. 37268899 PMC10239112

[ref29] Niżnik Ł.; Noga M.; Kobylarz D.; Frydrych A.; Krośniak A.; Kapka-Skrzypczak L.; Jurowski K. Gold Nanoparticles (AuNPs)—Toxicity, Safety and Green Synthesis: A Critical Review. Int. J. Mol. Sci. 2024, 25 (7), 4057 10.3390/ijms25074057. 38612865 PMC11012566

[ref30] Ccana-Ccapatinta G. V.; Mejía J. A. A.; Tanimoto M. H.; Groppo M.; de Carvalho J. C. A. S.; Bastos J. K. Dalbergia Ecastaphyllum (L.) Taub. And Symphonia Globulifera L.f. And Botanical Sources of Isoflavonoids and Benzophenones in Brazilian Red Propolis. Molecules 2020, 25 (9), 2060 10.3390/molecules25092060. 32354180 PMC7249054

[ref31] Aldana-Mejía J. A.; Ccana-Ccapatinta G. V.; Ribeiro V. P.; Arruda C.; Veneziani R. C. S.; Ambrósio S. R.; Bastos J. K. A Validated HPLC-UV Method for the Analysis of Phenolic Compounds in Brazilian Red Propolis and Dalbergia Ecastaphyllum. J. Pharm. Biomed. Anal. 2021, 198, 114029 10.1016/j.jpba.2021.114029. 33756382

[ref32] Botteon C. E. A.; Silva L. B.; Ccana-Ccapatinta G. V.; Silva T. S.; Ambrosio S. R.; Veneziani R. C. S.; Bastos J. K.; Marcato P. D. Biosynthesis and Characterization of Gold Nanoparticles Using Brazilian Red Propolis and Evaluation of Its Antimicrobial and Anticancer Activities. Sci. Rep. 2021, 11 (1), 1–16. 10.1038/s41598-021-81281-w. 33479338 PMC7820602

[ref33] Nguyen T. T.; Mammeri F.; Ammar S. Iron Oxide and Gold Based Magneto-Plasmonic Nanostructures for Medical Applications: A Review. Nanomaterials 2018, 8 (3), 149 10.3390/nano8030149. 29518969 PMC5869640

[ref34] Ghosh D.; Chattopadhyay N. Gold Nanoparticles: Acceptors for Efficient Energy Transfer from the Photoexcited Fluorophores. Opt. Photonics J. 2013, 03 (1), 18–26. 10.4236/opj.2013.31004.

[ref35] Wongsa P.; Phatikulrungsun P.; Prathumthong S. FT-IR Characteristics Phenolic Profiles and Inhibitory Potential against Digestive Enzymes of 25 Herbal Infusions. Sci. Rep. 2022, 12 (1), 6631 10.1038/s41598-022-10669-z. 35459897 PMC9033800

[ref36] do Nascimento T. G.; Redondo G. D. P.; de Araújo Abreu C. T.; Silva V. C.; Lira G. M.; Meireles Grillo L. A.; da Conceição M. M.; Freitas J. D.; Souza J. S.; Araújo Júnior J. X.; Basílio-Júnior I. D. Modified Release Microcapsules Loaded with Red Propolis Extract Obtained by Spray-Dryer Technique: Phytochemical, Thermal and Physicochemical Characterizations. J. Therm. Anal. Calorim. 2019, 138 (5), 3559–3569. 10.1007/s10973-019-08287-5.

[ref37] Silva V. C.; Silva A. M. G. S.; Basílio J. A. D.; Xavier J. A.; Do Nascimento T. G.; Naal R. M. Z. G.; Del Lama M. P.; Leonelo L. A. D.; Mergulhão N. L. O. N.; Maranhão F. C. A.; Silva D. M. W.; Owen R.; Duarte I. F. B.; Bulhões L. C. G.; Basílio I. D.; Goulart M. O. F. New Insights for Red Propolis of Alagoas—Chemical Constituents, Topical Membrane Formulations and Their Physicochemical and Biological Properties. Molecules 2020, 25 (24), 5811 10.3390/molecules25245811. 33317120 PMC7763695

[ref38] Sharaf S.; Higazy A.; Hebeish A. Propolis Induced Antibacterial Activity and Other Technical Properties of Cotton Textiles. Int. J. Biol. Macromol. 2013, 59, 408–416. 10.1016/j.ijbiomac.2013.04.030. 23665479

[ref39] Franca J. R.; De Luca M. P.; Ribeiro T. G.; Castilho R. O.; Moreira A. N.; Santos V. R.; Faraco A. A. G. Propolis - Based Chitosan Varnish: Drug Delivery, Controlled Release and Antimicrobial Activity against Oral Pathogen Bacteria. BMC Complement. Altern. Med. 2014, 14, 478 10.1186/1472-6882-14-478. 25495921 PMC4295328

[ref40] Oliveira R. N.; Mancini M. C.; Oliveira F. C. S. d.; Passos T. M.; Quilty B.; Thiré R. M. d. S. M.; McGuinness G. B. FTIR analysis and quantification of phenols and flavonoids of five commercially available plants extracts used in wound healing. Rev. Mater. 2016, 21 (3), 767–779. 10.1590/S1517-707620160003.0072.

[ref41] do Nascimento T. G.; da Silva P. F.; Azevedo L. F.; da Rocha L. G.; de Moraes Porto I. C. C.; Lima e Moura T. F. A.; Basílio-Júnior I. D.; Grillo L. A. M.; Dornelas C. B.; Fonseca E. J. da S.; de Jesus Oliveira E.; Zhang A. T.; Watson D. G. Polymeric Nanoparticles of Brazilian Red Propolis Extract: Preparation, Characterization, Antioxidant and Leishmanicidal Activity. Nanoscale Res. Lett. 2016, 11, 301 10.1186/s11671-016-1517-3. 27316742 PMC4912519

[ref42] Wolk A.; Rosenthal M.; Neuhaus S.; Huber K.; Brassat K.; Lindner J. K. N.; Grothe R.; Grundmeier G.; Bremser W.; Wilhelm R. A Novel Lubricant Based on Covalent Functionalized Graphene Oxide Quantum Dots. Sci. Rep. 2018, 8 (1), 1–9. 10.1038/s41598-018-24062-2. 29643400 PMC5895846

[ref43] Hegazi A. G.; El-Houssiny A. S.; Fouad E. A. Egyptian Propolis 14: Potential Antibacterial Activity of Propolis-Encapsulated Alginate Nanoparticles against Different Pathogenic Bacteria Strains. Adv. Nat. Sci. Nanosci. Nanotechnol. 2019, 10 (4), 045019 10.1088/2043-6254/ab52f4.

[ref44] Bharadwaj K. K.; Rabha B.; Pati S.; Sarkar T.; Choudhury B. K.; Barman A.; Bhattacharjya D.; Srivastava A.; Baishya D.; Edinur H. A.; Abdul Kari Z.; Mohd Noor N. H. Green Synthesis of Gold Nanoparticles Using Plant Extracts as Beneficial Prospect for Cancer Theranostics. Molecules 2021, 26 (21), 6389 10.3390/molecules26216389. 34770796 PMC8586976

[ref45] Patil S.; Chandrasekaran R. Biogenic Nanoparticles: A Comprehensive Perspective in Synthesis, Characterization, Application and Its Challenges. J. Genet. Eng. Biotechnol. 2020, 18 (1), 67 10.1186/s43141-020-00081-3. 33104931 PMC7588575

[ref46] Ghafariyan M. H.; Malakouti M. J.; Dadpour M. R.; Stroeve P.; Mahmoudi M. Effects of Magnetite Nanoparticles on Soybean Chlorophyll. Environ. Sci. Technol. 2013, 47 (18), 10645–10652. 10.1021/es402249b. 23951999

[ref47] Boros B. V.; Ostafe V. Evaluation of Ecotoxicology Assessment Methods of Nanomaterials and Their Effects. Nanomaterials 2020, 10 (4), 610 10.3390/nano10040610. 32224954 PMC7221575

[ref48] Miralles P.; Church T. L.; Harris A. T. Toxicity, Uptake, and Translocation of Engineered Nanomaterials in Vascular Plants. Environ. Sci. Technol. 2012, 46 (17), 9224–9239. 10.1021/es202995d. 22892035

[ref49] Verma D. K.; Patel S.; Kushwah K. S. Green Biosynthesis of Silver Nanoparticles and Impact on Growth, Chlorophyll, Yield and Phytotoxicity of Phaseolus Vulgaris L. Vegetos 2020, 33 (4), 648–657. 10.1007/s42535-020-00150-5.

[ref50] Bayat M.; Zargar M.; Murtazova K. M.-S.; Nakhaev M. R.; Shkurkin S. I. Ameliorating Seed Germination and Seedling Growth of Nano-Primed Wheat and Flax Seeds Using Seven Biogenic Metal-Based Nanoparticles. Agronomy 2022, 12 (4), 811 10.3390/agronomy12040811.

[ref51] M S A.; Sridharan K.; Puthur J. T.; Dhankher O. P. Priming with Nanoscale Materials for Boosting Abiotic Stress Tolerance in Crop Plants. J. Agric. Food Chem. 2021, 69 (35), 10017–10035. 10.1021/acs.jafc.1c03673. 34459588

[ref52] Chandrasekaran U.; Luo X.; Wang Q.; Shu K. Are There Unidentified Factors Involved in the Germination of Nanoprimed Seeds?. Front. Plant Sci. 2020, 11, 1–6. 10.3389/fpls.2020.00832. 32587599 PMC7298061

[ref53] Maraghni M.; Gorai M.; Neffati M. Seed Germination at Different Temperatures and Water Stress Levels, and Seedling Emergence from Different Depths of Ziziphus Lotus. South African J. Bot. 2010, 76 (3), 453–459. 10.1016/j.sajb.2010.02.092.

[ref54] Asli S.; Neumann P. M. Colloidal Suspensions of Clay or Titanium Dioxide Nanoparticles Can Inhibit Leaf Growth and Transpiration via Physical Effects on Root Water Transport. Plant, Cell Environ. 2009, 32 (5), 577–584. 10.1111/j.1365-3040.2009.01952.x. 19210640

[ref55] Arora S.; Sharma P.; Kumar S.; Nayan R.; Khanna P. K.; Zaidi M. G. H. Gold-Nanoparticle Induced Enhancement in Growth and Seed Yield of Brassica Juncea. Plant Growth Regul. 2012, 66 (3), 303–310. 10.1007/s10725-011-9649-z.

[ref56] Mahakham W.; Theerakulpisut P.; Maensiri S.; Phumying S.; Sarmah A. K. Environmentally Benign Synthesis of Phytochemicals-Capped Gold Nanoparticles as Nanopriming Agent for Promoting Maize Seed Germination. Sci. Total Environ. 2016, 573, 1089–1102. 10.1016/j.scitotenv.2016.08.120. 27639594

[ref57] Pandey A. C.; Sanjay S. S.; Yadav R. S. Application of ZnO Nanoparticles in Influencing the Growth Rate of Cicer Arietinum. J. Exp. Nanosci. 2010, 5 (6), 488–497. 10.1080/17458081003649648.

[ref58] Venzhik Y.; Deryabin A.; Popov V.; Dykman L.; Moshkov I. Priming with Gold Nanoparticles Leads to Changes in the Photosynthetic Apparatus and Improves the Cold Tolerance of Wheat. Plant Physiol. Biochem. 2022, 190, 145–155. 10.1016/j.plaphy.2022.09.006. 36115268

[ref59] Das S.; Debnath N.; Pradhan S.; Goswami A. Enhancement of Photon Absorption in the Light-Harvesting Complex of Isolated Chloroplast in the Presence of Plasmonic Gold Nanosol—a Nanobionic Approach towards Photosynthesis and Plant Primary Growth Augmentation. Gold Bull. 2017, 50 (3), 247–257. 10.1007/s13404-017-0214-z.

[ref60] Iavicoli I.; Leso V.; Fontana L.; Calabrese E. J. Nanoparticle Exposure and Hormetic Dose–Responses: An Update. Int. J. Mol. Sci. 2018, 19 (3), 805 10.3390/ijms19030805. 29534471 PMC5877666

[ref61] Ye Y.; Cota-Ruiz K.; Gardea-Torresdey J. L. Plant-Nano Interactions: Lessons Learned from 15 Years of Nanophytotoxicity Studies; Academic Press, 2023; pp 275–292.

[ref62] Zuverza-Mena N.; Armendariz R.; Peralta-Videa J. R.; Gardea-Torresdey J. L. Effects of Silver Nanoparticles on Radish Sprouts: Root Growth Reduction and Modifications in the Nutritional Value. Front. Plant Sci. 2016, 7, 90 10.3389/fpls.2016.00090. 26909084 PMC4754487

[ref63] Lin D.; Xing B. Root Uptake and Phytotoxicity of ZnO Nanoparticles. Environ. Sci. Technol. 2008, 42 (15), 5580–5585. 10.1021/es800422x. 18754479

[ref64] Alidoust D.; Isoda A. Phytotoxicity Assessment of γ-Fe2O3 Nanoparticles on Root Elongation and Growth of Rice Plant. Environ. Earth Sci. 2014, 71 (12), 5173–5182. 10.1007/s12665-013-2920-z.

[ref65] Deng F.; Wang S.; Xin H. Toxicity of CuO Nanoparticles to Structure and Metabolic Activity of Allium Cepa Root Tips. Bull. Environ. Contam. Toxicol. 2016, 97 (5), 702–708. 10.1007/s00128-016-1934-0. 27704188

[ref66] Sun Y.; Jing R.; Zheng F.; Zhang S.; Jiao W.; Wang F. Evaluating Phytotoxicity of Bare and Starch-Stabilized Zero-Valent Iron Nanoparticles in Mung Bean. Chemosphere 2019, 236, 124336 10.1016/j.chemosphere.2019.07.067. 31310976

[ref67] Shelar A.; Nile S. H.; Singh A. V.; Rothenstein D.; Bill J.; Xiao J.; Chaskar M.; Kai G.; Patil R. Recent Advances in Nano-Enabled Seed Treatment Strategies for Sustainable Agriculture: Challenges, Risk Assessment, and Future Perspectives. Nano-Micro Lett. 2023, 15 (1), 54 10.1007/s40820-023-01025-5. PMC9935810 36795339

[ref68] Zhang Y.; Martinez M. R.; Sun H.; Sun M.; Yin R.; Yan J.; Marelli B.; Giraldo J. P.; Matyjaszewski K.; Tilton R. D.; Lowry G. V. Charge, Aspect Ratio, and Plant Species Affect Uptake Efficiency and Translocation of Polymeric Agrochemical Nanocarriers. Environ. Sci. Technol. 2023, 57 (22), 8269–8279. 10.1021/acs.est.3c01154. 37227395 PMC10249409

[ref69] Guo H.; Liu Y.; Chen J.; Zhu Y.; Zhang Z. The Effects of Several Metal Nanoparticles on Seed Germination and Seedling Growth: A Meta-Analysis. Coatings 2022, 12 (2), 183 10.3390/coatings12020183.

[ref70] Coman V.; Oprea I.; Leopold L. F.; Vodnar D. C.; Coman C. Soybean Interaction with Engineered Nanomaterials: A Literature Review of Recent Data. Nanomaterials 2019, 9 (9), 1248 10.3390/nano9091248. 31484310 PMC6780927

[ref71] Yoon S. J.; Kwak J. I.; Lee W. M.; Holden P. A.; An Y. J. Zinc Oxide Nanoparticles Delay Soybean Development: A Standard Soil Microcosm Study. Ecotoxicol. Environ. Saf. 2014, 100 (1), 131–137. 10.1016/j.ecoenv.2013.10.014. 24296285

[ref72] Ajiboye T. T.; Ajiboye T. O.; Babalola O. O. Impacts of Binary Oxide Nanoparticles on the Soybean Plant and Its Rhizosphere, Associated Phytohormones, and Enzymes. Molecules 2023, 28 (3), 1326 10.3390/molecules28031326. 36770994 PMC9919940

[ref73] Mitra D.; Mondal R.; Khoshru B.; Shadangi S.; Das Mohapatra P. K.; Panneerselvam P. Rhizobacteria Mediated Seed Bio-Priming Triggers the Resistance and Plant Growth for Sustainable Crop Production. Curr. Res. Microb. Sci. 2021, 2, 100071 10.1016/j.crmicr.2021.100071. 34841361 PMC8610296

[ref74] Habibi N.; Aryan S.; Amin M. W.; Sanada A.; Terada N.; Koshio K. Potential Benefits of Seed Priming under Salt Stress Conditions on Physiological, and Biochemical Attributes of Micro-Tom Tomato Plants. Plants 2023, 12 (11), 2187 10.3390/plants12112187. 37299165 PMC10255691

[ref75] Nile S. H.; Thiruvengadam M.; Wang Y.; Samynathan R.; Shariati M. A.; Rebezov M.; Nile A.; Sun M.; Venkidasamy B.; Xiao J.; Kai G. Nano-Priming as Emerging Seed Priming Technology for Sustainable Agriculture—Recent Developments and Future Perspectives. J. Nanobiotechnology 2022, 20, 254 10.1186/s12951-022-01423-8. 35659295 PMC9164476

[ref76] GuhaSarkar S.; Banerjee R. Intravesical Drug Delivery: Challenges, Current Status, Opportunities and Novel Strategies. J. Controlled Release 2010, 148 (2), 147–159. 10.1016/j.jconrel.2010.08.031. 20831887

[ref77] Ma X.; Geiser-Lee J.; Deng Y.; Kolmakov A. Interactions between Engineered Nanoparticles (ENPs) and Plants: Phytotoxicity, Uptake and Accumulation. Sci. Total Environ. 2010, 408 (16), 3053–3061. 10.1016/j.scitotenv.2010.03.031. 20435342

[ref78] Dietz K. J.; Mittler R.; Noctor G. Recent Progress in Understanding the Role of Reactive Oxygen Species in Plant Cell Signaling. Plant Physiol. 2016, 171 (3), 1535–1539. 10.1104/pp.16.00938. 27385820 PMC4936595

[ref79] Mittler R. ROS Are Good. Trends Plant Sci. 2017, 22 (1), 11–19. 10.1016/j.tplants.2016.08.002. 27666517

[ref80] Salachna P.; Byczyńska A.; Zawadzińska A.; Piechocki R.; Mizielińska M. Stimulatory Effect of Silver Nanoparticles on the Growth and Flowering of Potted Oriental Lilies. Agronomy 2019, 9 (10), 610 10.3390/agronomy9100610.

[ref81] Thiruvengadam M.; Gurunathan S.; Chung I. M. Physiological, Metabolic, and Transcriptional Effects of Biologically-Synthesized Silver Nanoparticles in Turnip (Brassica Rapa Ssp. Rapa L.). Protoplasma 2015, 252 (4), 1031–1046. 10.1007/s00709-014-0738-5. 25471476

[ref82] Baskar V.; Venkatesh J.; Park S. W. Impact of Biologically Synthesized Silver Nanoparticles on the Growth and Physiological Responses in Brassica Rapa Ssp. Pekinensis. Environ. Sci. Pollut. Res. 2015, 22 (22), 17672–17682. 10.1007/s11356-015-4864-1. 26154034

[ref83] Thakur R. K.; Dhirta B.; Shirkot P. Studies on Effect of Gold Nanoparticles on Meloidogyne Incognita and Tomato Plants Growth and Development. bioRxiv 2018, 428144 10.1101/428144.

[ref84] Rajeshwari A.; Suresh S.; Chandrasekaran N.; Mukherjee A. Toxicity Evaluation of Gold Nanoparticles Using an Allium Cepa Bioassay. RSC Adv. 2016, 6 (29), 24000–24009. 10.1039/C6RA04712B. PMC13353057 42433444

[ref85] Li H.; Ye X.; Guo X.; Geng Z.; Wang G. Effects of Surface Ligands on the Uptake and Transport of Gold Nanoparticles in Rice and Tomato. J. Hazard. Mater. 2016, 314, 188–196. 10.1016/j.jhazmat.2016.04.043. 27131459

[ref86] Siegel J.; Záruba K.; Švorčík V.; Kroumanová K.; Burketová L.; Martinec J. Round-Shape Gold Nanoparticles: Effect of Particle Size and Concentration on Arabidopsis Thaliana Root Growth. Nanoscale Res. Lett. 2018, 13, 95 10.1186/s11671-018-2510-9. 29637317 PMC5893504

[ref87] Amooaghaie R.; Saeri M. R.; Azizi M. Synthesis Characterization and Biocompatibility of Silver Nanoparticles Synthesized from Nigella Sativa Leaf Extract in Comparison with Chemical Silver Nanoparticles. Ecotoxicol. Environ. Saf. 2015, 120, 400–408. 10.1016/j.ecoenv.2015.06.025. 26122733

[ref88] Shirley B. W. Flavonoids in Seeds and Grains: Physiological Function, Agronomic Importance and the Genetics of Biosynthesis. Seed Sci. Res. 1998, 8 (4), 415–422. 10.1017/S0960258500004372.

[ref89] Song C.; Xiang D. B.; Yan L.; Song Y.; Zhao G.; Wang Y. H.; Zhang B. L. Changes in Seed Growth, Levels and Distribution of Flavonoids during Tartary Buckwheat Seed Development. Plant Prod. Sci. 2016, 19 (4), 518–527. 10.1080/1343943X.2016.1207485.

[ref90] Treutter D. Significance of Flavonoids in Plant Resistance: A Review. Environ. Chem. Lett. 2006, 4 (3), 147–157. 10.1007/s10311-006-0068-8.

[ref91] Shah A.; Smith D. L. Flavonoids in Agriculture: Chemistry and Roles in, Biotic and Abiotic Stress Responses, and Microbial Associations. Agronomy 2020, 10 (8), 1209 10.3390/agronomy10081209.

[ref92] Shah A.; Subramanian S.; Smith D. L. Seed Priming with Devosia Sp. Cell-Free Supernatant (CFS) and Citrus Bioflavonoids Enhance Canola and Soybean Seed Germination. Molecules 2022, 27 (11), 3410 10.3390/molecules27113410. 35684348 PMC9182190

[ref93] Graham T. L. Flavonoid and Isoflavonoid Distribution in Developing Soybean Seedling Tissues and in Seed and Root Exudates. Plant Physiol. 1991, 95 (2), 594–603. 10.1104/pp.95.2.594. 16668024 PMC1077573

[ref94] Weston L. A.; Mathesius U. Flavonoids: Their Structure, Biosynthesis and Role in the Rhizosphere, Including Allelopathy. J. Chem. Ecol. 2013, 39 (2), 283–297. 10.1007/s10886-013-0248-5. 23397456

[ref95] Palma-Tenango M.; Soto-Hernández M.; Aguirre-Hernández E. Flavonoids in Agriculture. Flavonoids—From Biosynthesis to Human Health; IntechOpen, 2017. 10.5772/intechopen.68626.

[ref96] Tang J.; Shen H.; Zhang R.; Yang F.; Hu J.; Che J.; Dai H.; Tong H.; Wu Q.; Zhang Y.; Su Q. Seed Priming with Rutin Enhances Tomato Resistance against the Whitefly Bemisia Tabaci. Pestic. Biochem. Physiol. 2023, 194, 105470 10.1016/j.pestbp.2023.105470. 37532344

[ref97] Aldana-Mejia J. A.; Ccana-Ccapatinta G. V.; Squarisi I. S.; Nascimento S.; Tanimoto M. H.; Ribeiro V. P.; Arruda C.; Nicolella H.; Esperandim T.; Ribeiro A. B.; De Freitas K. S.; Da Silva L. H. D.; Ozelin S. D.; Oliveira L. T. S.; Melo A. L. A.; Tavares D. C.; Bastos J. K. Nonclinical Toxicological Studies of Brazilian Red Propolis and Its Primary Botanical Source Dalbergia Ecastaphyllum. Chem. Res. Toxicol. 2021, 34 (4), 1024–1033. 10.1021/acs.chemrestox.0c00356. 33720704

[ref98] Bar-Ilan O.; Albrecht R. M.; Fako V. E.; Furgeson D. Y. Toxicity Assessments of Multisized Gold and Silver Nanoparticles in Zebrafish Embryos. Small 2009, 5 (16), 1897–1910. 10.1002/smll.200801716. 19437466

[ref99] Bai C.; Tang M. Toxicological Study of Metal and Metal Oxide Nanoparticles in Zebrafish. J. Appl. Toxicol. 2020, 40 (1), 37–63. 10.1002/jat.3910. 31884684

[ref100] Sadhukhan S.; Moniruzzaman M.; Maity S.; Ghosh S.; Pattanayak A. K.; Chakraborty S. B.; Maity B.; Das M. Organometallic Folate Gold Nanoparticles Ameliorate Lipopolysaccharide-Induced Oxidative Damage and Inflammation in Zebrafish Brain. ACS Omega 2022, 7 (11), 9917–9928. 10.1021/acsomega.2c00415. 35350341 PMC8945078

[ref101] Chung Y. C.; Chen I. H.; Chen C. J. The Surface Modification of Silver Nanoparticles by Phosphoryl Disulfides for Improved Biocompatibility and Intracellular Uptake. Biomaterials 2008, 29 (12), 1807–1816. 10.1016/j.biomaterials.2007.12.032. 18242693

[ref102] Krishnaraj C.; Young G. M.; Yun S. Il. In Vitro Embryotoxicity and Mode of Antibacterial Mechanistic Study of Gold and Copper Nanoparticles Synthesized from Angelica Keiskei (Miq.) Koidz. Leaves Extract. Saudi J. Biol. Sci. 2022, 29 (4), 2552–2563. 10.1016/j.sjbs.2021.12.039. 35531254 PMC9072899

[ref103] Machado S.; González-Ballesteros N.; Gonçalves A.; Magalhães L.; de Passos M. S. P.; Rodríguez-Argüelles M. C.; Gomes A. C. Toxicity in Vitro and in Zebrafish Embryonic Development of Gold Nanoparticles Biosynthesized Using Cystoseira Macroalgae Extracts. Int. J. Nanomedicine 2021, 16, 5017–5036. 10.2147/IJN.S300674. 34326639 PMC8315781

[ref104] Ahmad T.; Bustam M. A.; Irfan M.; Moniruzzaman M.; Asghar H. M. A.; Bhattacharjee S. Mechanistic Investigation of Phytochemicals Involved in Green Synthesis of Gold Nanoparticles Using Aqueous Elaeis Guineensis Leaves Extract: Role of Phenolic Compounds and Flavonoids. Biotechnol. Appl. Biochem. 2019, 66 (4), 698–708. 10.1002/bab.1787. 31172593

[ref105] Fierascu I.; Georgiev M. I.; Ortan A.; Fierascu R. C.; Avramescu S. M.; Ionescu D.; Sutan A.; Brinzan A.; Ditu L. M. Phyto-Mediated Metallic Nano-Architectures via Melissa Officinalis L.: Synthesis, Characterization and Biological Properties. Sci. Rep. 2017, 7 (1), 12428 10.1038/s41598-017-12804-7. 28963525 PMC5622205

[ref106] Bugel S. M.; Bonventre J. A.; Tanguay R. L. Comparative Developmental Toxicity of Flavonoids Using an Integrative Zebrafish System. Toxicol. Sci. 2016, 154 (1), 55–68. 10.1093/toxsci/kfw139. 27492224 PMC5091365

[ref107] Daugsch A.; Moraes C. S.; Fort P.; Park Y. K. Brazilian Red Propolis - Chemical Composition and Botanical Origin. Evidence-based Complement. Altern. Med. 2008, 5 (4), 435–441. 10.1093/ecam/nem057. PMC2586321 18955226

[ref108] da Silva L. H. D.; Squarisi I. S.; de Freitas K. S.; Barcelos Ribeiro A.; Ozelin S. D.; Aldana-Mejía J. A.; de Oliveira L. T. S.; Rodrigues T. E.; de Melo M. R. S.; Nicolella H. D.; Alves B. S.; de Andrade Melo A. L.; Ccana-Ccapatinta G. V.; Bastos J. K.; Tavares D. C. Toxicological and Chemoprevention Studies of Dalbergia Ecastaphyllum (L.) Taub. Stem, the Botanical Source of Brazilian Red Propolis. J. Pharm. Pharmacol. 2022, 74 (5), 740–749. 10.1093/jpp/rgac008. 35299250

[ref109] Kristofco L. A.; Haddad S. P.; Chambliss C. K.; Brooks B. W. Differential Uptake of and Sensitivity to Diphenhydramine in Embryonic and Larval Zebrafish. Environ. Toxicol. Chem. 2018, 37 (4), 1175–1181. 10.1002/etc.4068. 29274281

[ref110] Cheng J.; Flahaut E.; Cheng S. H. Effect of Carbon Nanotubes on Developing Zebrafish (Danio Rerio) Embryos. Environ. Toxicol. Chem. 2007, 26 (4), 708–716. 10.1897/06-272R.1. 17447555

[ref111] Pitt J. A.; Kozal J. S.; Jayasundara N.; Massarsky A.; Trevisan R.; Geitner N.; Wiesner M.; Levin E. D.; Di Giulio R. T. Uptake, Tissue Distribution, and Toxicity of Polystyrene Nanoparticles in Developing Zebrafish (Danio Rerio). Aquat. Toxicol. 2018, 194, 185–194. 10.1016/j.aquatox.2017.11.017. 29197232 PMC6959514

[ref112] Duan Z.; Duan X.; Zhao S.; Wang X.; Wang J.; Liu Y.; Peng Y.; Gong Z.; Wang L. Barrier Function of Zebrafish Embryonic Chorions against Microplastics and Nanoplastics and Its Impact on Embryo Development. J. Hazard. Mater. 2020, 395, 122621 10.1016/j.jhazmat.2020.122621. 32289630

[ref113] Luís L. G.; Ferreira P.; Fonte E.; Oliveira M.; Guilhermino L. Does the Presence of Microplastics Influence the Acute Toxicity of Chromium(VI) to Early Juveniles of the Common Goby (Pomatoschistus Microps)? A Study with Juveniles from Two Wild Estuarine Populations. Aquat. Toxicol. 2015, 164, 163–174. 10.1016/j.aquatox.2015.04.018. 26004740

[ref114] Sant K. E.; Timme-Laragy A. R. Zebrafish as a Model for Toxicological Perturbation of Yolk and Nutrition in the Early Embryo. Curr. Environ. Heal. reports 2018, 5 (1), 125–133. 10.1007/s40572-018-0183-2. PMC5876134 29417450

[ref115] Hill A. J.; Bello S. M.; Prasch A. L.; Peterson R. E.; Heideman W. Water Permeability and TCDD-Induced Edema in Zebrafish Early-Life Stages. Toxicol. Sci. 2004, 78 (1), 78–87. 10.1093/toxsci/kfh056. 14718644

[ref116] Souder J. P.; Gorelick D. A. Quantification of Estradiol Uptake in Zebrafish Embryos and Larvae. Toxicol. Sci. 2017, 158 (2), 465–474. 10.1093/toxsci/kfx107. 28535311 PMC5837308

[ref117] Dolgova N. V.; Hackett M. J.; MacDonald T. C.; Nehzati S.; James A. K.; Krone P. H.; George G. N.; Pickering I. J. Distribution of Selenium in Zebrafish Larvae after Exposure to Organic and Inorganic Selenium Forms. Metallomics 2016, 8 (3), 305–312. 10.1039/C5MT00279F. 26781816

[ref118] Assémat E.; Vinot S.; Gofflot F.; Linsel-Nitschke P.; Illien F.; Châtelet F.; Verroust P.; Louvet-Vallée S.; Rinninger F.; Kozyraki R. Expression and Role of Cubilin in the Internalization of Nutrients during the Peri-Implantation Development of the Rodent Embryo. Biol. Reprod. 2005, 72 (5), 1079–1086. 10.1095/biolreprod.104.036913. 15616221

[ref119] Halbach K.; Ulrich N.; Goss K. U.; Seiwert B.; Wagner S.; Scholz S.; Luckenbach T.; Bauer C.; Schweiger N.; Reemtsma T. Yolk Sac of Zebrafish Embryos as Backpack for Chemicals?. Environ. Sci. Technol. 2020, 54 (16), 10159–10169. 10.1021/acs.est.0c02068. 32639148

[ref120] Zainol Abidin I. Z.; Fazry S.; Jamar N. H.; Ediwar Dyari H. R.; Zainal Ariffin Z.; Johari A. N.; Ashaari N. S.; Johari N. A.; Megat Abdul Wahab R.; Zainal Ariffin S. H. The Effects of Piper Sarmentosum Aqueous Extracts on Zebrafish (Danio Rerio) Embryos and Caudal Fin Tissue Regeneration. Sci. Rep. 2020, 10 (1), 1–11. 10.1038/s41598-020-70962-7. 32843675 PMC7447815

[ref121] Tran M. H.; Nguyen T. V. A.; Do H. G.; Kieu T. K.; Nguyen T. K. T.; Le H. D.; Guerrero-Limon G.; Massoz L.; Nivelle R.; Zappia J.; Pham H. T.; Nguyen L. T.; Muller M. Testing Biological Actions of Medicinal Plants from Northern Vietnam on Zebrafish Embryos and Larvae: Developmental, Behavioral, and Putative Therapeutical Effects. PLoS ONE 2023, 18, e0294048 10.1371/journal.pone.0294048. 37934745 PMC10629648

[ref122] Verma S. K.; Jha E.; Panda P. K.; Kumari P.; Pramanik N.; Kumari S.; Thirumurugan A. Molecular Investigation to RNA and Protein Based Interaction Induced in Vivo Biocompatibility of Phytofabricated AuNP with Embryonic Zebrafish. Artif. Cells Nanomed. Biotechnol. 2018, 46, 671–684. 10.1080/21691401.2018.1505746. 30311784

[ref123] Wiegand M. D. Composition, Accumulation and Utilization of Yolk Lipids in Teleost Fish. Rev. Fish Biol. Fish. 1996, 6 (3), 259–286. 10.1007/BF00122583.

[ref124] Raldúa D.; André M.; Babin P. J. Clofibrate and Gemfibrozil Induce an Embryonic Malabsorption Syndrome in Zebrafish. Toxicol. Appl. Pharmacol. 2008, 228 (3), 301–314. 10.1016/j.taap.2007.11.016. 18358510

[ref125] Kuder R. S.; Gundala H. P. Developmental Toxicity of Deltamethrin and 3-Phenoxybenzoic Acid in Embryo–Larval Stages of Zebrafish (Danio Rerio). Toxicol. Mech. Methods 2018, 28 (6), 415–422. 10.1080/15376516.2018.1439131. 29421951

[ref126] Qiang L.; Arabeyyat Z. H.; Xin Q.; Paunov V. N.; Dale I. J. F.; Lloyd Mills R. I.; Rotchell J. M.; Cheng J. Silver Nanoparticles in Zebrafish (Danio Rerio) Embryos: Uptake, Growth and Molecular Responses. Int. J. Mol. Sci. 2020, 21 (5), 1876 10.3390/ijms21051876. 32182933 PMC7084859

[ref127] Song Z.; Zhang Y.; Zhang H.; Rajendran R. S.; Wang R.; Hsiao C. D.; Li J.; Xia Q.; Liu K. Isoliquiritigenin Triggers Developmental Toxicity and Oxidative Stress–Mediated Apoptosis in Zebrafish Embryos/Larvae via Nrf2-HO1/JNK-ERK/Mitochondrion Pathway. Chemosphere 2020, 246, 125727 10.1016/j.chemosphere.2019.125727. 31896010

[ref128] Oberdörster G.; Oberdörster E.; Oberdörster J. Nanotoxicology: An Emerging Discipline Evolving from Studies of Ultrafine Particles. Environ. Health Perspect. 2005, 113 (7), 823–839. 10.1289/ehp.7339. 16002369 PMC1257642

[ref129] Kumar V.; Guleria P.; Kumar V.; Yadav S. K. Gold Nanoparticle Exposure Induces Growth and Yield Enhancement in Arabidopsis Thaliana. Sci. Total Environ. 2013, 461–462, 462–468. 10.1016/j.scitotenv.2013.05.018. 23747561

[ref130] OECD. Test No. 236: Fish Embryo Acute Toxicity (FET) Test: OECD Guidelines for the Testing of Chemicals, Section 2; OECD Publishing: Paris, 2013.

